# Dual role of the receptor kinase FERONIA in regulating tissue mechanics and growth

**DOI:** 10.1126/sciadv.aeb8608

**Published:** 2026-07-15

**Authors:** Elise Muller, Marc Ropitaux, Gaëlle Durambur, Simone Bovio, Valentin Laplaud, Leon Gebhard, Arnaud Lehner, Stéphanie Drevensek, Arezki Boudaoud

**Affiliations:** ^1^ Laboratoire d’ Hydrodynamique, Ecole Polytechnique, 91120 Palaiseau, France.; ^2^ Université de Rouen Normandie, GLYCOMEV UR 4358, SFR Normandie Végétal FED 4277, Innovation Chimie Carnot, IRIB, F-76000 Rouen, France.; ^3^ Université de Rouen Normandie, INSERM, CNRS, HeRacLeS US51 UAR2026, PRIMACEN, F-76000 Rouen, France.; ^4^ Laboratoire Reproduction et Développement des Plantes, ENS de Lyon, 69007 Lyon, France.; ^5^ PLATIM-LyMIC, University of Lyon, SFR Biosciences, ENS de Lyon, INSERM US8, CNRS UMS3444, UCBL, 50 Avenue Tony Garnier, 69007 Lyon, France.

## Abstract

During morphogenesis, growing organisms adapt to internal and external mechanical stress. In plants, the receptor kinase FERONIA is involved in a broad range of responses to perturbations of the cell wall, but it is unclear which cues are sensed and how this sensing controls growth. Here, we addressed these questions in *Marchantia* vegetative propagules. We combined culture in microfluidic chips to quantify growth and mechanical properties of propagules, perturbations with osmotic treatments, characterization of cell wall polysaccharides, and a mathematical model of cell wall expansion that incorporates sensing. We found that FERONIA independently regulates tissue properties that govern elastic deformation and growth rates. We propose that the regulation of growth by the FERONIA pathway relies on both a positive feedback from elastic deformation of the wall and a negative feedback from wall expansion. Together, we expect our quantitative framework to be broadly relevant to the investigation of how mechanical cues guide development.

## Teaser

The receptor kinase FERONIA mediates a double feedback from tissue mechanics on growth rate.

## INTRODUCTION

The growth of a plant cell is driven by a high inner pressure, known as turgor pressure, and is limited by the surrounding cell wall, a stiff extracellular matrix mostly made of intertwined polysaccharides. Turgor-generated cell wall tension is relaxed by remodeling of the polysaccharide network or by integration of new polysaccharides (*1*, *2*). Relaxation notably involves cell wall responses to the mechanical stress associated with tension. Responses may be “passive,” corresponding to elastic stretching of the polysaccharides or to plastic (irreversible) deformation arising from polysaccharides sliding along each other (*3*). However, the robustness and adaptability of plant growth in a fluctuating environment require “active” responses, which may be triggered by local mechanical stress or strain (*4*). Forces applied to cell walls are perceived by several classes of mechanosensors and/or sensors of cell wall integrity. Here, we focus on the role in growth of *FERONIA*, a salient member of the *Catharanthus roseus* receptor-like kinase 1-like (*Cr*RLK1L) family. On the one hand, its role in the regulation of growth was widely characterized, and several upstream and downstream signaling pathways were described, involving, in particular, cell wall–related effectors. On the other hand, those extensive descriptions do not allow to fully understand how *FERONIA* regulates growth (*5*, *6*). Moreover, *FERONIA* is a signaling node involved in fertility, immunity, responses to biotic and abiotic stress, as well as in growth and development (*7*), which makes it difficult to study its specific function in growth.

Growth control by *FERONIA* was characterized in various organs and under environmental conditions, leading to the conclusion that *FERONIA* is a context-dependent regulator (*8*). On the one hand, *FERONIA* may inhibit growth, as demonstrated by *Arabidopsis feronia* mutants (At*fer*), which display overgrowth of the pollen tube within the embryo sac (*9*) or bigger seeds (*10*). The secreted peptide rapid alkalinization factor (RALF) activates AtFERONIA to suppresses cell elongation (*11*) or to inhibit vacuolar expansion (*12*). On the other hand, *FERONIA* may promote growth, as shown by the reduced size of At*fer* hypocotyls (*13*, *14*), of rosettes (*15*, *16*), of trichomes, or of root hairs (*16*), although the presence of FERONIA was also demonstrated to allow dynamical root hair growth inhibition by the small peptide RALF22 (*17*). Mechanosensing by *FERONIA* was also found to limit growth fluctuation in *Arabidopsis* roots (*18*). The role of *FERONIA* in growth regulation seems to be evolutionary conserved in land plant (*19*). A first study in the model liverwort *Marchantia polymorpha* reported a reduced thallus (vegetative body) in *feronia* mutants (Mp*fer*) (*19*).

Cell wall integrity sensors such as FERONIA mediate responses to cell wall damage (*20*). The nature of the activating signal and downstream signaling are still under investigation. Integrity sensors are mainly known to bind to wall-derived signaling molecules or to cell wall components, but may also act as a scaffold for cell wall proteins and peptides. In particular, FERONIA binds to RALF peptides (*7*, *11*) and to pectins (preferentially de-methylesterified pectins) (*21*, *22*). FERONIA interacts with co-receptors of the LRE-LIKE GPI-AP family (LLG1 and LLG2) (*6*) and forms complexes with the extracellular LEUCINE-RICH REPEAT EXTENSIN (*5*, *23*), which are also involved in cell wall integrity sensing. FERONIA responds to pathogen-associated molecular patterns, leading to cross-talks with immunity (*24*). It was also hypothesized that integrity sensors could act as true mechanosensors and respond to deformations of the membrane–cell wall continuum (*25*). This hypothesis is supported by reduced calcium signaling in At*fer* (*18*) upon mechanical bending and by the partial rescue of At*fer* by osmotic treatments to reduce mechanical stress (*26*). To assess what mechanical signals may activate FERONIA and how this contributes to growth regulation, we combined modeling and experiments. The use of modeling has been instrumental to identify rules underlying the alignment of cortical microtubules in aerial organs (*27*–*29*) or strain-stiffening during seed growth (*30*).

After activation, FERONIA can trigger several downstream signaling pathways. It modifies calcium signaling, reactive oxygen species (ROS) production, apoplasmic pH, hormonal signaling, actin regulation, and vesicular trafficking, in addition to transcriptional regulation (*6*, *31*). In particular, FERONIA controls cell wall composition. At*fer* mutants have a reduced cellulose content and alteration in matrix monosaccharide composition (*32*). FERONIA is also involved in the regulation of pectin methylesterification and of pectin cross-linking by calcium ions (*21*, *33*). Accordingly, many phenotypes of At*fer* mutants point to an alteration of the mechanical properties of cells and tissues (*5*). At*fer* mutants show reduced apoplasmic pH (*11*) and altered lignin composition (*34*). Cell bursting in tip-growing cells (*16*, *19*) and in diffusely growing pavement cells (*26*) indicates weaker cell wall or higher turgor in *fer* mutants, compared to wild type (WT). Indentation-based measurements show lower apparent modulus in At*fer* roots subject to salt stress and in Mp*fer* thalli, suggesting weaker cell wall or lower turgor than in WT. A mutant of another *Cr*RLK1L member, *THESEUS*, displays a decreased Brillouin elastic contrast (*35*). Here, we independently quantified turgor pressure and tissue elasticity to fully assess their regulation by *FERONIA*.

Together, we aimed at building a quantitative framework to study the role of *FERONIA* in the regulation of tissue mechanics and growth, which led us to identify potential mechanical signals that may activate *FERONIA*. We took advantage of many features of *M. polymorpha*. The *Cr*RLK1L has a single member Mp*FER*. *Marchantia* may reproduce vegetatively by producing a large number of genetically identical gemmae. Following immersion in water, gemmae germinate and start to grow (*36*) and can be cultivated in microfluidic chips (*37*). We thus investigated the role of *FERONIA* in gemmae growth.

## RESULTS

### *FERONIA* promotes and patterns growth in *Marchantia* gemmae

**Fig. 1.**
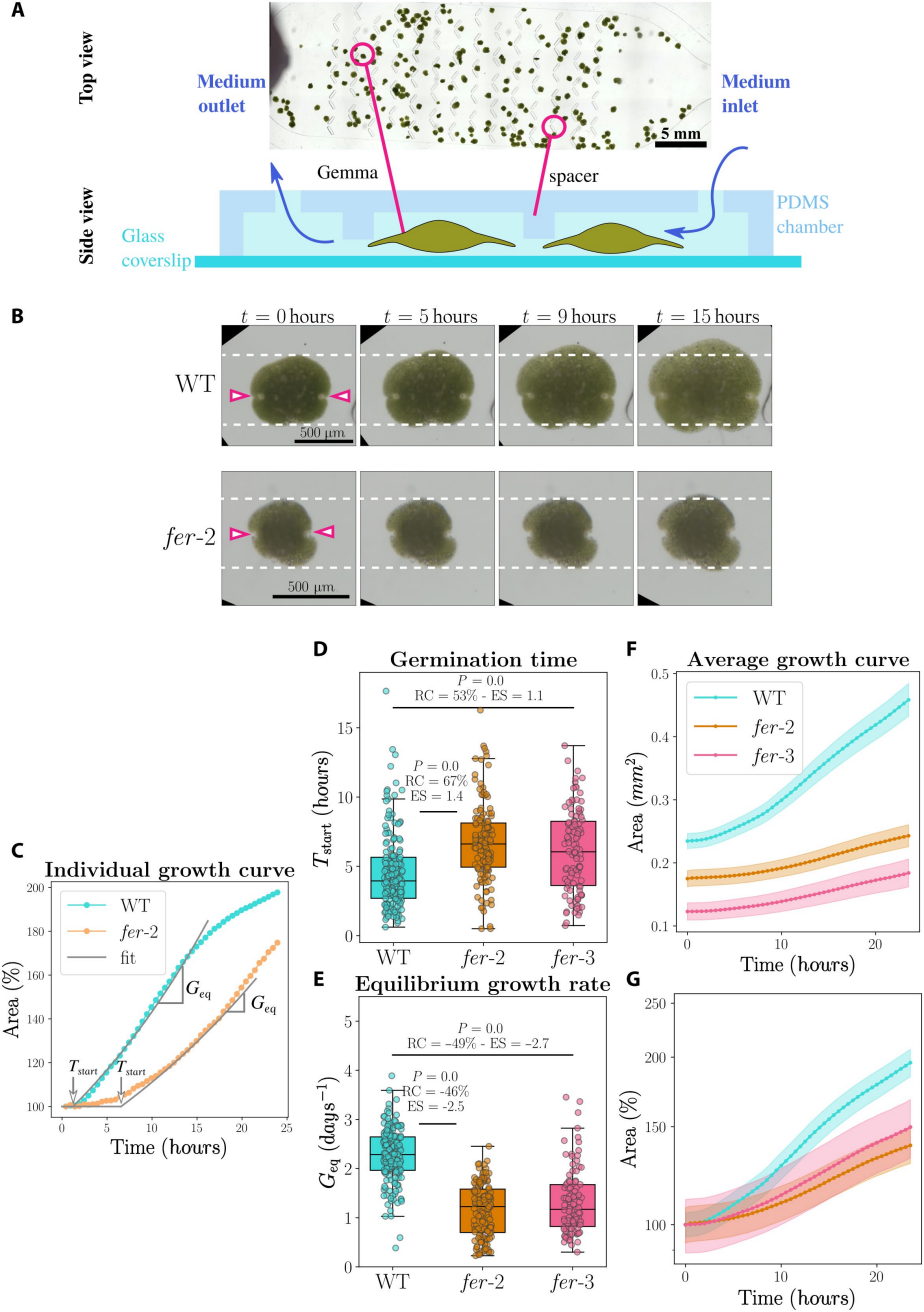
FERONIA promotes gemmae growth. (**A**) Microfluidic chip to grow gemmae. Top: Top view of a microfluidic chip filled with gemmae. Medium inlet and outlet are on the sides. One gemma and one trap (spacer) are indicated with pink circles. Scale bar, 5 mm. Bottom: Sketch of the chip side view; one gemma and one spacer are indicated by pink lines. The (slow) flow of the culture medium is represented by blue arrows. (**B**) Bright-field images of representative growing gemmae of WT and *fer*-2 at relevant time points (0, 5, 9, and 15 hours after imbibition, i.e.,immersion in water). Meristematic regions (notches) are indicated by pink arrow heads. The dotted white lines are guides to observe gemmae growth. Scale bars, 500 μm. (**C**) Representative individual growth curve of WT and *fer*-2 over the first 24 hours of growth after imbibition. The gray lines are the delayed exponential fit for each genotype. Characteristic parameters for growth are represented on the curves: the germination starting time *T*_start_ and the equilibrium growth rate *G*_eq_. (**D** to **F**) Growth of parameters of WT and mutants *fer*-2 and *fer*-3. [(D) and (E)] Box plot and scatter plot of the germination starting time *T*_start_ (D) and equilibrium growth rate *G*_eq_ (E). The top and bottom of the box correspond to the lower and upper quartiles. (F) Average absolute area as a function of time. (**G**) Average area (relative to initial area) as a function of time. [(F) and (G)] The shaded areas correspond to the 95% confidence interval. WT: *n* = 178 individuals and rep. = 5 replicates. *fer*-2: *n* = 141 and rep. = 3. *fer*-3: *n* = 104 and rep. = 3.

Previous work showed that Mp*fer* mutants have reduced cell and thallus sizes after several days or weeks of growth (*19*). To test whether this expands to early development, we recorded the first day of gemmae life in a microfluidic chip continuously supplied with liquid growth medium. The chip consists of a polydimethylsiloxane (PDMS) growth chamber, decorated with spacers—to isolate individual gemmae—and sealed with a glass slide (Fig. 1, A and B); we found that gemmae grow similarly in chips and outside of chips (*37*). Gemmae growth is quantified based on area. It can be divided into two phases based on the parametrization of growth curve. A first rest phase shows almost no growth and lasts for a duration *T*_start_ of a few hours (1 to 10 hours), spanning imbibition (uptake of water upon immersion of gemmae) to germination (start of growth). The ensuing growth phase is characterized by an equilibrium growth rate *G*_eq_, with some fluctuations around this average (Fig. 1C). Two independent Mp*fer* knockout mutant lines, *fer*-2 and *fer*-3 (*19*), exhibit both a late germination and a low equilibrium growth rate, compared to WT (Fig. 1, D and E). Two independent Mp*FER* overexpressor lines, *FER*ox-5 and *FER*ox-9, (*19*) exhibit opposite trends, with an early germination and a higher equilibrium growth rate, compared to WT (fig. S1, A to D). Thus, *FERONIA* promotes germination and growth, at gemma scale.

**Fig. 2.**
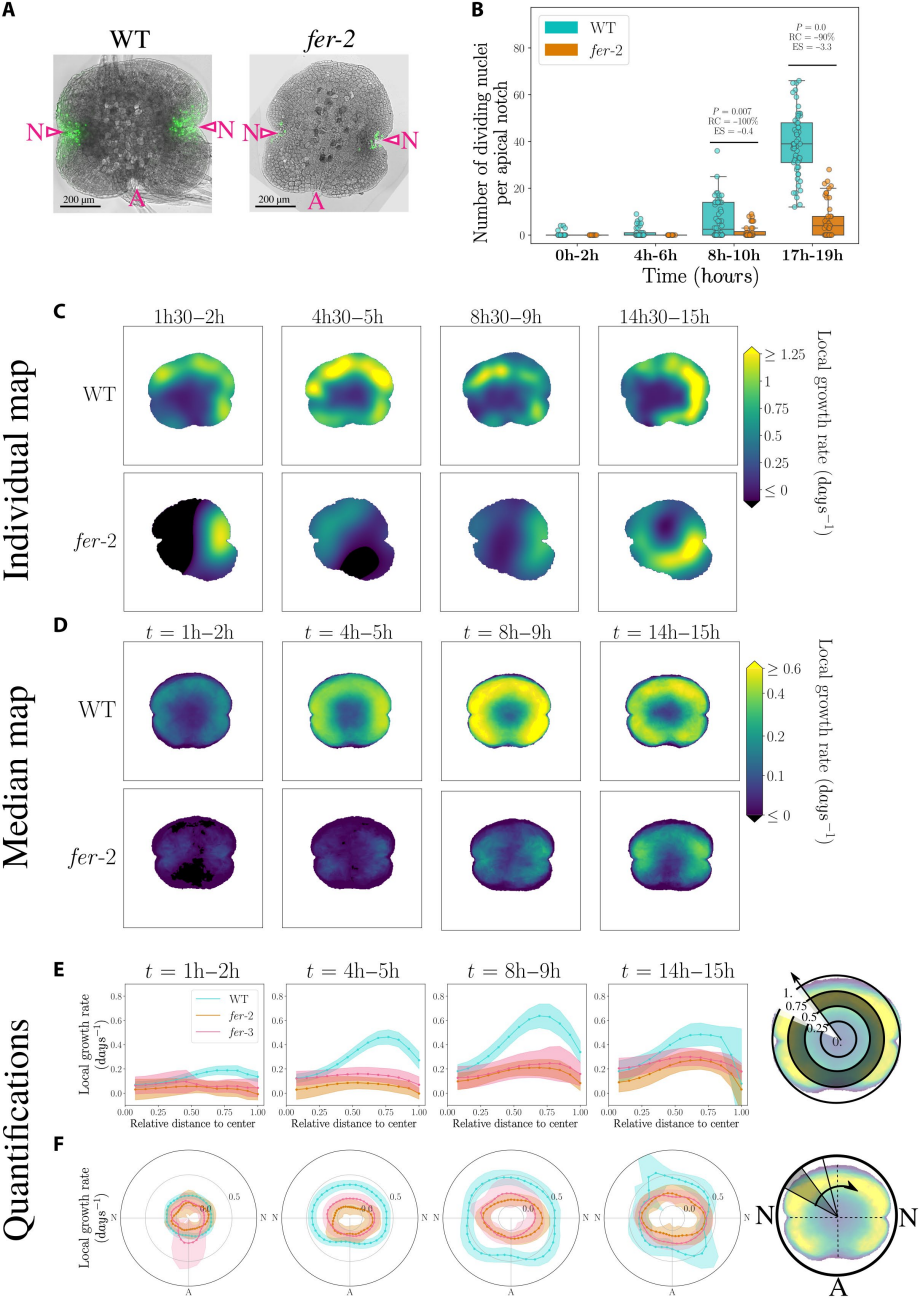
*FERONIA* regulates proliferation and local growth patterning. (**A** and **B**) Quantification of proliferation in WT and *fer*-2. (A) Representative merge confocal images of fluorescence intensity from EdU staining (green) and of bright-field gemma view (gray) at 17 to 19 hours. Meristematic regions (notches) are indicated by pink arrow heads and letter N, while the former attachment point to the mother plant is indicated by the letter A. Scale bars, 200 μm. (B) Box plot and scatter plot of the number of dividing nuclei (stained with EdU) per notch. WT: *n*(0h to 2h) = 10, *n*(4h to 6h) = 24, *n*(8h to 10h) = 27, *n*(17h to 19h) = 29, rep. = 2. *fer*-2: *n*(0h to 2h) = 10, *n*(4h to 6h) = 18, *n*(8h to 10h) = 27, *n*(17h to 19h) = 29, rep. = 2. (**C**) Local growth rate maps for representative individual gemmae of WT and *fer*-2. Local growth rate is quantified between two imaging time points (given after imbibition) and is represented with a linear color scale. (**D**) Median local growth rate maps for WT and *fer*-2. The median local growth rate is calculated over the individuals and over 1 hour at different time interval (after imbibition). It is represented with a symmetric logarithmic color scale. (**E** and **F**) Radial and circumferential quantification of the local growth rate for WT, *fer*-2, and *fer*-3. (E) Mean local growth rate of the gemma surface at a given distance from the center of the gemmae, averaged over all orientations (schematic on the right). (F) Mean local growth rate of the gemma surface in a given angular sector, averaged over all distances to the center (schematic on the right). The shaded areas indicate the 95% confidence interval. WT: *n* = 61, rep. = 3. *fer*-2: *n* = 57, rep. = 3 for (D) to (F). h, hours.

Next, we investigated whether *FERONIA* controls spatial differences in growth rate, notably because different functional domains make up a gemma. For instance, each gemma normally contains two meristematic regions known as apical notches (*38*) (highlighted by pink arrows in Fig. 1B), from which more differentiated tissues are derived during gemma development. To isolate the contribution of proliferation from that of elongation, the number of dividing cells was assessed by S-phase nuclei stained using 5-ethynyl-2′-deoxyuridine (EdU) incorporation at different time points after imbibition. Proliferation is restricted to apical notches, consistent with classical descriptions (*38*) (pink arrows in Fig. 2A). In WT, proliferation can start as early as 2 hours after imbibition, while first dividing nuclei in *fer*-2 are present only from 8 to 10 hours after imbibition. Moreover, even after proliferation begins in *fer*-2 gemmae, we still observe a higher number of dividing nuclei in WT than in *fer*-2 (Fig. 2B). The large difference in proliferation between WT and *fer*-2 cannot be explained by gemmae area, which is about 25% smaller in *fer*-2 (Fig. 1F). Moreover, no cell death was observed as early as 1 or 8 hours after removal from the cup (fig. S2, A, B, D, and E), contrary to later stages in *fer*-2 (fig. S2F) (*19*). Accordingly, *FERONIA* promotes early timing and high rate of proliferation.

We then considered the contribution of *FERONIA* to the regulation of elongation. Local, supracellular growth rate maps were generated by computing local displacement on growing gemmae using optical images taken every 30 min with a stereomicroscope [see Materials and Methods]. Growth maps feature substantial spatiotemporal variability (Fig. 2C), although systematic differences between center and periphery of gemmae can be presumed. To reveal such differences, we averaged the growth maps of about 60 individuals [see Materials and Methods] at different key time points (1 to 2 hours, which is before germination, 4 to 5 hours at WT germination, 8 to 9 hours at *fer*-2 germination, and 14 to 15 hours after germination) (Fig. 2D). Starting from germination, a “growth ring” appears on WT gemmae (4- to 5-hour time point), the intensity of which tends to increase (8- to 9-hour time point) and finally reduces as the global growth rate diminishes (Fig. 2D). To assess radial patterns of growth, we averaged local growth rate at a given distance from the center of the gemma. Following germination, this radial average indeed shows a peak of growth rate at a relative distance of 75% from the center (Fig. 2E). For *fer*-2 and *fer*-3, the growth ring is almost absent (see Fig. 2, C and D, and radial quantification in Fig. 2E). In the mutants, growth rate is maximal in the meristematic regions, appearing less affected there (Fig. 2D). To assess circumferential patterns of growth, we averaged local growth rate at a given angle with respect to the line joining notches. This angular average is indeed higher at the angles corresponding to apical notches for *fer*-2 and *fer*-3 (Fig. 2F). Angular averaging also shows that the local growth rate is diminished in *fer*-2 and *fer*-3 compared to WT, except at the angle of the apical notches, where mutants and WT local growth rates are the closest (Fig. 2, D and F). We note in particular that the growth rate of *fer*-2 and *fer*-3 is reduced in regions without proliferation, meaning that *FERONIA* controls cell expansion. The spatial pattern of growth does not seem stronger in the overexpression lines but rather well defined earlier than in WT (fig. S1, E to G), suggesting that patterning requires *FERONIA*, but that its strength is not *FERONIA* dose-dependent. Together, *FERONIA* regulates and patterns growth by independently promoting proliferation and elongation.

### *FERONIA* regulates cell wall polysaccharide composition

**Fig. 3.**
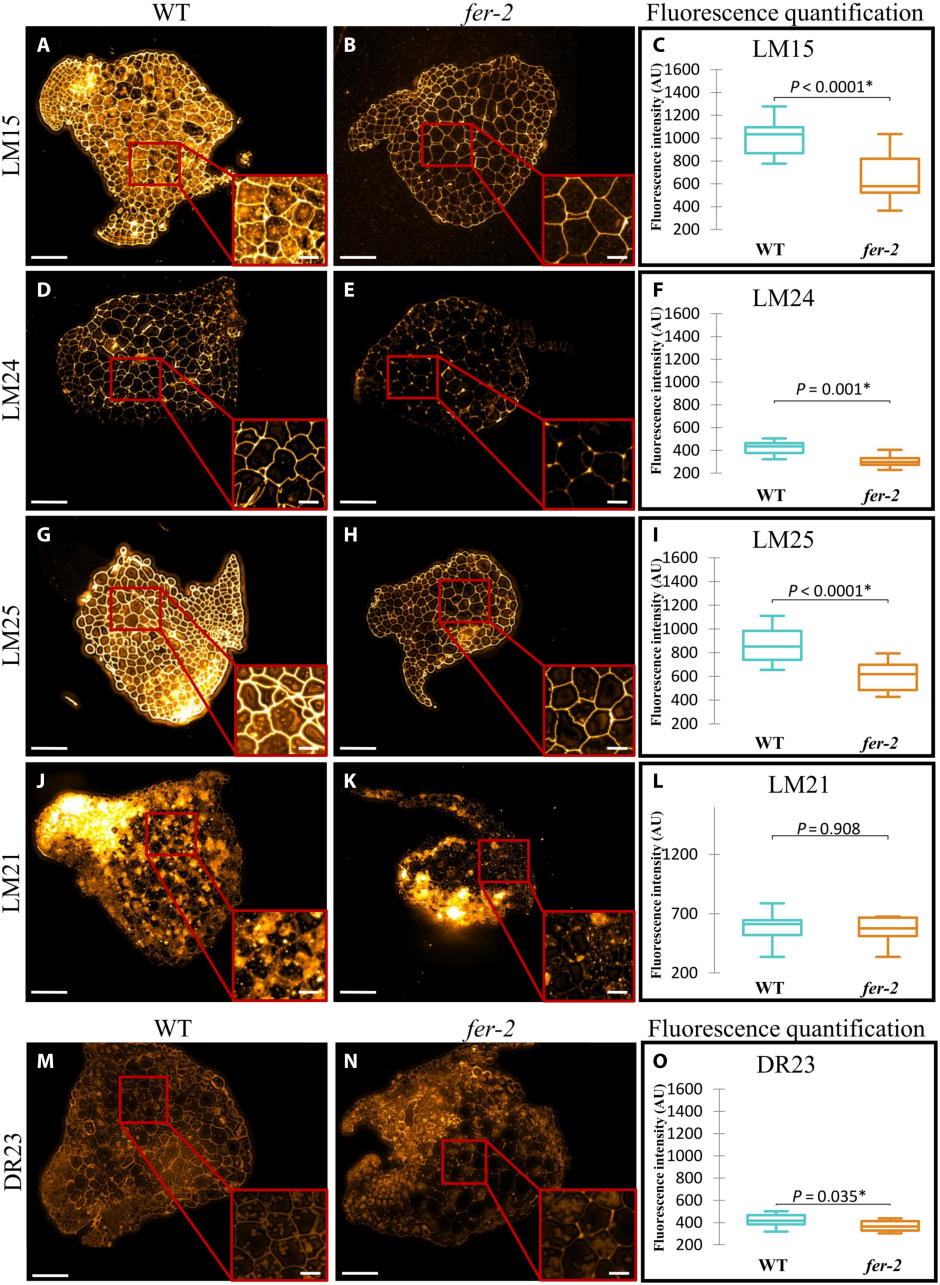
*FERONIA* affects cell wall hemicellulosic polymers distribution. (**A**, **D**, **G**, **J**, and **M**) Immunocytochemical labeling of WT and (**B**, **E**, **H**, **K**, and **N**) *fer*-2 mutant line gemmae at 8 hours after imbibition. All samples were fixed, dehydrated, embedded, and cut (2-μm thickness) before immunolabeling with primary antibodies: LM15 [xylosylated xyloglucan (XXXG)], LM24 [galactosylated xyloglucan (XXLG)], LM25 [galactosylated xyloglucan (XXLG, XLLG)], LM21 [β-(1→4)-mannan backbone epitope from heteromannan], and DR23 (cellulose microfibrils). (**C**, **F**, **I**, **L**, and **O**) Box plots illustrating the fluorescence intensity. Each histogram represents the means of 14 biological replicates. Mann-Whitney tests were used; the star (*) represents the significant difference at α = 0.05. Scale bars, 100 μm (main images) and 25 μm (magnified images). AU, arbitrary unit.

We next sought how FERONIA may regulate elongation. FERONIA affects cell wall composition in cellulose and matrix polysaccharides in *Arabidopsis* (*32*). The cell wall is thus a good candidate to mediate the growth phenotypes observed in *Marchantia fer* mutants. We probed polysaccharide distributions in the cell wall of gemmae using immunolabeling of classical representatives of different classes of polysaccharides: hemicelluloses (and, notably, xyloglucans) and pectins that are part of the matrix polysaccharides, as well as cellulose (monoclonal antibodies and their recognized epitopes are listed in table S2). Tested polysaccharides are chosen among those shown to be present in *Marchantia*’s cell wall (*39*), which are shown to display strong similarities with other known plant cell walls, except that the pectic fraction is reduced, especially in older tissues (*40*). We detected a decrease in the fluorescence associated to both the xyloglucan backbone (∼−35% LM15; Fig. 3, A to C) and galactosylated side chains [∼−24% LM24 (Fig. 3, D to F); ∼−30% LM25 (Fig. 3, G to I)] in *fer*-2 mutant compared to WT. As *Marchantia* does not have xyloglucan fucosylated side chain, we did not use the CCRC-M1 antibody recognizing those patterns (*41*). Moreover, among hemicelluloses, signal reduction seems to be specific to xyloglucan, as the other hemicelluloses tested, heteromannan with LM21, have similar labeling pattern and level of fluorescence between WT and *fer*-2 (Fig. 3, J to L). These results suggest changes either in xyloglucan content or in xyloglucan accessibility in the cell wall, which might explain part of the reduction of growth in *fer*-2.

We further tested whether signal associated to Direct Red 23 (DR23) was affected in *fer*-2 and showed that associated fluorescent signal is decreased in *fer*-2 (∼−12% DR23; Fig. 3, J to L). DR23 labels cellulose but also xyloglucan (*42*), so at least part of the observed signal reduction can be ascribed to the reduction associated to xyloglucan, making it difficult to conclude about changes in cellulose content in *fer*-2.

**Fig. 4.**
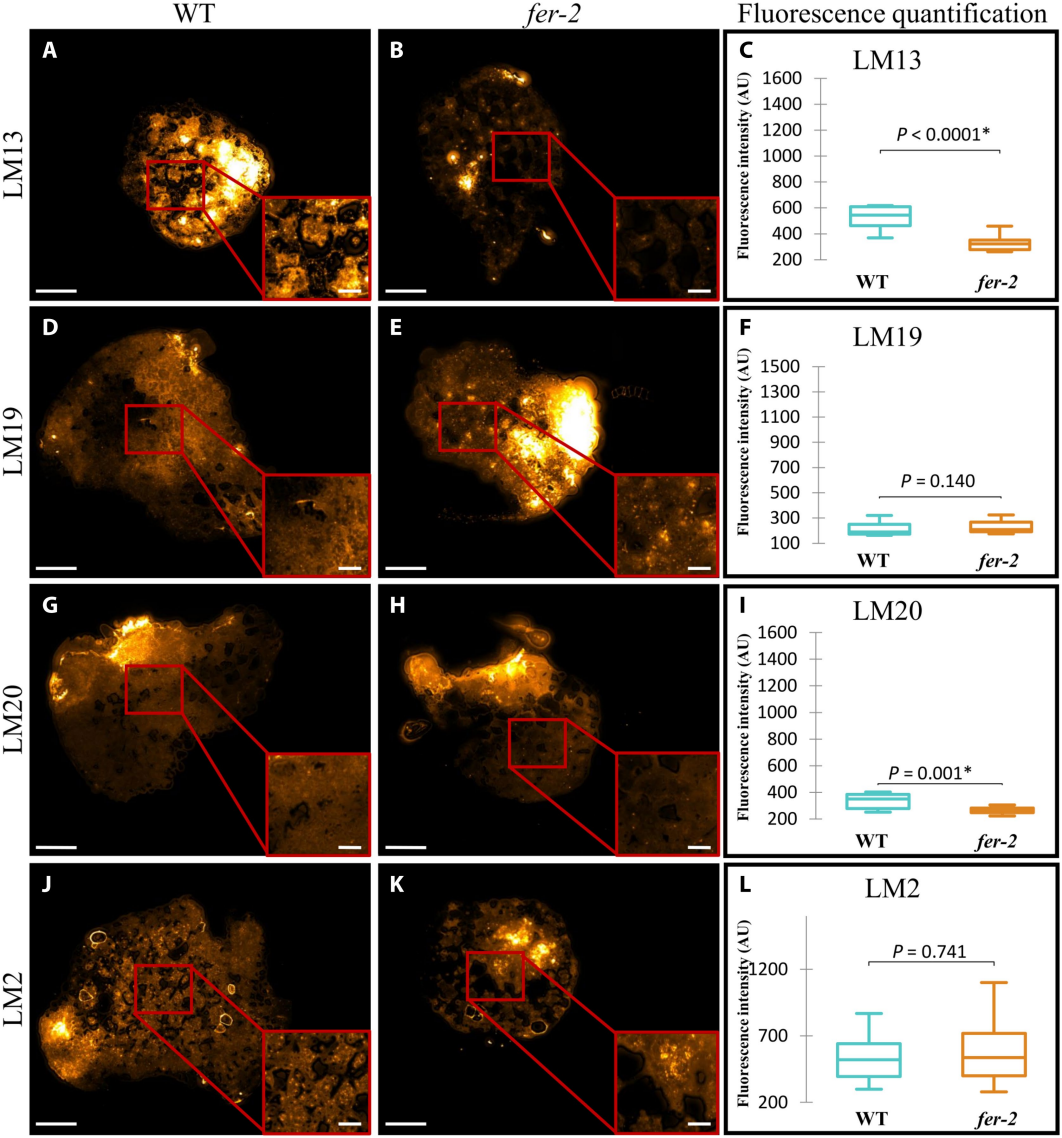
Cell wall pectin domains and arabinogalactan proteins distribution and quantification of fluorescence intensity. (**A**, **D**, **G**, and **J**) Immunocytochemical labeling of WT and (**B**, **E**, **H**, and **K**) *fer*-2 mutant line gemmae at 8 hours after imbibition. All samples were fixed, dehydrated, embedded, and cut (2-μm thickness) before immunolabeling with primary antibodies: LM13 [specific subset of unbranched pectic (1→5)-α-l-arabinan], LM19 (homogalacturonan with low degree of esterification), LM20 (homogalacturonan with high degree of esterification), and LM2 [(1→6)-β-d-galactan chain with terminally attached glucuronic acid]. (**C**, **F**, **I**, and **L**) Box plots illustrating the fluorescence intensity. Each histogram represents the means of 14 biological replicates. Mann-Whitney tests were used, and * represents a significant difference at α = 0.05. The up and down sides of the box are the lower and upper quartiles. Scale bars, 100 μm (main images) and 25 μm (magnified images).

We characterized the pectic fraction of *fer*-2 cell wall. Immunolocalization using LM13, a monoclonal antibody recognizing rhamnogalacturonan-I with unbranched arabinan side chains, showed a strong reduction of the signal (∼−42% LM13; Fig. 4, A to C). The fluorescence associated with LM19 (low methylesterified homogalacturonan) immunolabeling was unchanged in *fer*-2 compared to WT (LM19; Fig. 4, D to F), while fluorescence associated to LM20 (highly esterified homogalacturonan) was reduced (∼−21% LM20; Fig. 4, G to I). This shows that *fer*-2 cell wall contains overall less pectins and less esterified homogalacturonan. Last, the signal associated to arabinogalactan proteins is unchanged (LM2; Fig. 4, J to L).

Together, these data suggest that FERONIA is involved in synthesis, delivery, or remodeling of xyloglucans and pectins (and possibly cellulose), potentially explaining growth control by FERONIA. Because cell wall mechanical properties depend on cell wall composition, we next examined whether FERONIA controls gemmae mechanics and whether this could regulate growth.

### *FERONIA* promotes and patterns gemmae turgor and stiffness, independently of growth regulation

**Fig. 5.**
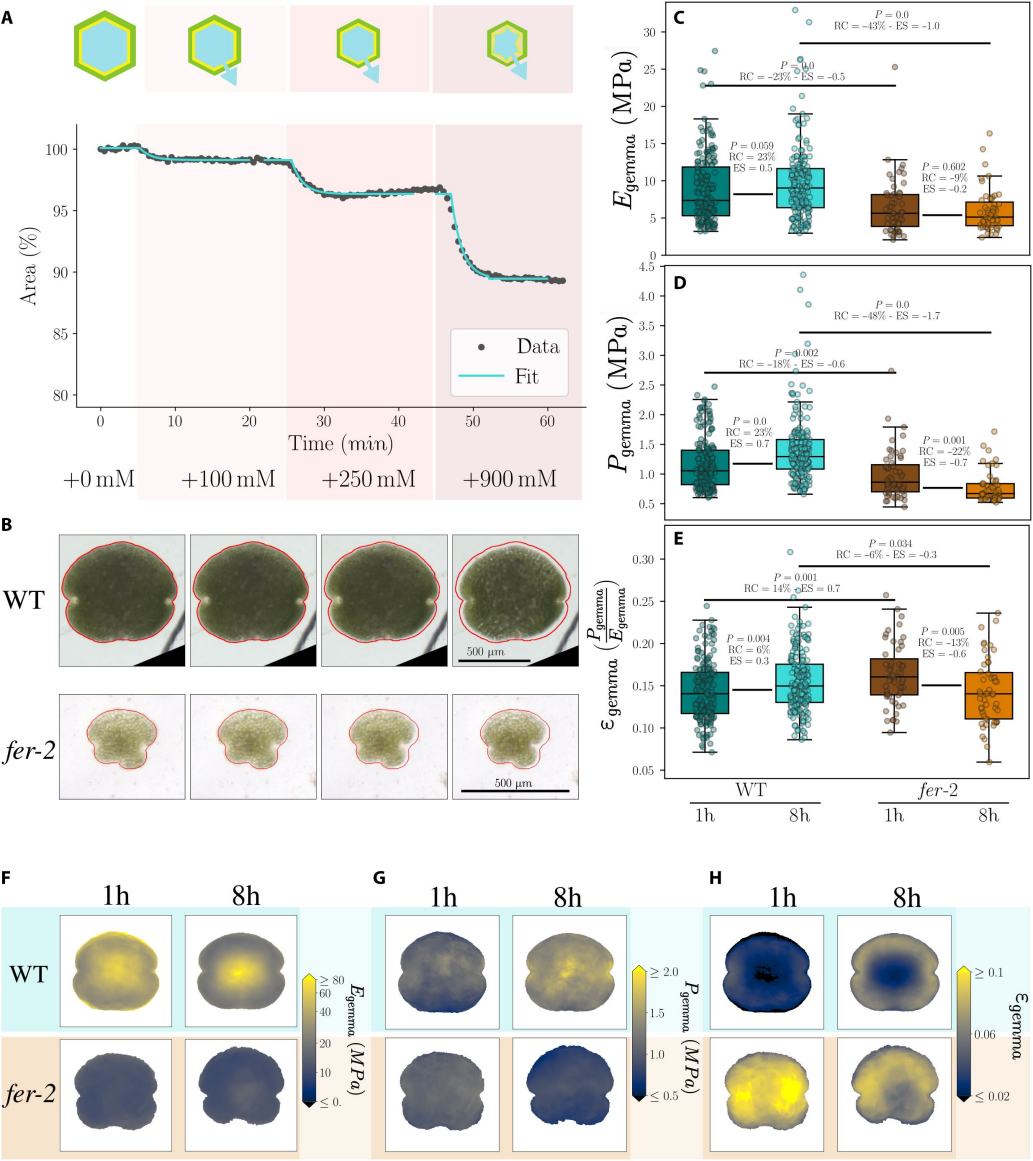
*FERONIA* regulates mechanical properties and their patterning. (**A**) Area of a gemma (% of initial area before osmotic steps) throughout an assay with several osmotic steps and fit of the predicted exponential decay to extract equilibrium values. Gemmae are subjected to three successive steps of +100, +250, and +900 mM (the last leading to plasmolysis) of mannitol. The top panel is a schematic of cell behaviors under these steps. The blue arrow represents net water flux. (**B**) Representative WT and *fer*-2 gemmae during osmotic steps (+0, +100, +250, and +900 mM). The red line is the initial contour of the gemma for each genotype and can be used as a visual guide for subsequent area reduction. Scale bars, 500 μm. (**C** to **E**) Box plot and scatter plot of measured mechanical properties at the gemma scale for WT and *fer*-2 gemmae at 1 and 8 hours after imbibition. (C) Volumetric elastic modulus *E*_gemma_, (D) turgor pressure *P*_gemma_, and (E) elastic deformation $\varepsilon_{\text{gemma}}=P_{\text{gemma}}/E_{\text{gemma}}$. WT: *n*(1h) = 176, *n*(8h) = 193, rep. = 4. *fer*-2: *n*(1h) = 55, *n*(8h) = 51, rep. = 3. (**F** to **H**) Maps of local mechanical parameters for WT and *fer*-2 gemmae at 1 and 8 hours after imbibition. Parameters are estimated locally from the median displacement during the osmotic steps. (F) Maps of local elastic modulus *E*_gemma_ with a symmetric logarithmic color scale, (G) maps of turgor *P*_gemma_ with a linear color scale, and (H) maps of the elastic deformation, ε_gemma_ with a linear color scale. WT: *n*(1h) = 142, *n*(8h) = 151, rep. = 3. *fer*-2: *n*(1h) = 41, *n*(8h) = 32, rep. = 3.

In previous work, the lack of FERONIA activity was found to affect cell or tissue mechanics (*16*, *19*, *21*), providing a potential explanation for growth defects in mutants. We thus wondered whether *fer* mutants gemmae exhibit mechanical defects. To address this question, we quantified the mechanical properties of WT and *fer*-2 gemmae, before germination (at 1 hour after imbibition) and after (at 8 hours after imbibition). We measured gemma elastic two-dimensional volumetric modulus (*E*_gemma_) and turgor pressure (*P*_gemma_) using osmotic steps rapidly applied to the gemma in chip (Fig. 5, A and B). Equilibrium areas following each step are extracted, and mechanical properties are deduced from the equilibration of water potential. As a given equilibrium depends on both turgor and stiffness, the analysis of two equilibria allows us to separate the contribution of the stiffness and of turgor to observed deformations [mechanical model detailed in Materials and methods]. As gemmae display the previously described strain-stiffening behavior of plant walls—meaning that gemmae walls are stiffer as the deformation from the rest state increases (*43*)—different osmotic intensities were used to improve the determination of mechanical parameters (Fig. 5A) [Materials and methods]. Figure 5 (C and D) shows our results. First, *fer*-2 gemmae are softer than WT. Temporal variation of stiffness seems also affected by the lack of *FERONIA*, as a slight increase of the volumetric elastic modulus is observable in the WT between 1 and 8 hours, while the elastic modulus remains constant for *fer*-2 (Fig. 5C). Similarly, *fer*-2 gemmae have a lower turgor pressure compared to WT, which tends to decrease after germination while it is maintained in WT (Fig. 5D). So, at gemma scale, *FERONIA* promotes stiffness and a build-up of turgor.

We also used a microindenter to apply a perpendicular force on plasmolyzed gemmae and obtained indentation moduli from their center and from their periphery. Similar with osmotic shifts, we found that *fer*-2 gemmae are softer. However, we did not detect temporal variations with this approach (fig. S3, A and B). This difference between osmotic shift and indentation could be due to either the difference in the type of loading (multidirectional with osmotic shift and perpendicular with indentation) or to the lower number of samples associated with a low throughput method.

**Fig. 6.**
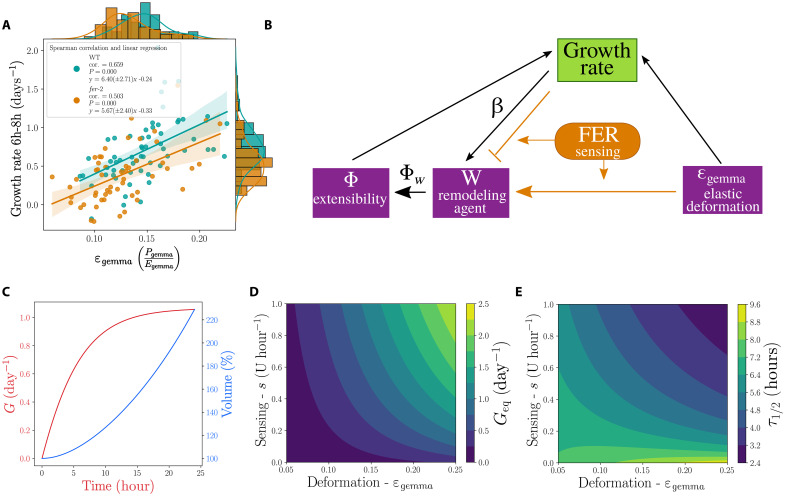
A mathematical model predicts the regulation of extensibility by FERONIA. (**A**) Correlation analysis of the instantaneous growth rate averaged between 6 and 8 hours post-imbibition with the elastic deformation ε_gemma_ for WT and *fer*-2. Scatter plot and regression line with 95% confidence interval are represented as well as histograms per genotype for each variable. Correlations are quantified by the Spearman correlation coefficient (cor.) and a linear model is fitted to the data, in which parameters are given with the 95% confidence interval. WT: *n* = 62, rep. = 3. *fer*-2: *n* = 56, rep. = 3. (**B**) Schematic representation of the minimal mathematical model, describing the relationship between the growth rate, the elastic deformation (ε_gemma_), the unknown remodeling agent *W*, its effect on extensibility (Φ) through the molar extensibility Φ*_w_* and *FERONIA* sensing, as well as the dependence on the autocatalytic rate β. (**C**) Instantaneous growth rate *G* and volume *V* over time as calculated by the model for $k_{w}^{+}$ = 0.01 U hour^−1^, $k_{w}^{-}$ = 0.1 hour^−1^, *s* = 0.9 U hour^−1^, *t*_s_ = 1 hour, β=0.5 U, P=1.5 MPa, E=10 MPa, and Y=0.01. (**D** and **E**) Phase diagram of the calculated growth parameters according to the toy model depending on sensing intensity *s* and on deformation ε_gemma_, for $k_{w}^{+}$ = 0.01 U hour^−1^, $k_{w}^{-}=0.1\;{\text{hour}}^{-1}$, $t_{s}=1\;\text{hour}$, β=0.5 U, E=10 MPa, and Y=0.01. (D) Calculated equilibrium growth rate *G*_eq_ and (E) and germination time τ_1/2_.

Next, we investigated whether such mechanical perturbation could be the cause of the growth deregulation in *fer* mutants. We tested whether gemmae at 8 hours after imbibition grow in accordance with Lockhart’s law (*44*), according to which cell growth rate increases linearly with turgor pressure. Unexpectedly, gemma growth rate is not positively correlated to turgor (fig. S4A). Our data rather show that growth rate negatively correlates with the volumetric elastic modulus *E*_gemma_, for both WT and *fer*-2 (fig. S4B). We previously observed the same negative correlation at 30 hours after imbibition, which leads to the hypothesis that, for gemmae, the elastic deformation $\varepsilon_{\text{gemma}}=P_{\text{gemma}}/E_{\text{gemma}}$ is the relevant mechanical factor to analyze growth (*37*). In our 8 hours gemma dataset, global growth rate is indeed positively correlated with elastic deformation ε_gemma_, this correlation being stronger than with *P*_gemma_ or *E*_gemma_ individually (Fig. 6A). We thus considered the following equation to describe gemma growth

$$G=\frac{1}{V}\frac{dV}{dt}=\Phi_{\text{eff}}\left(\varepsilon_{\text{gemma}}-y\right)$$ (1)

which defines the instantaneous growth rate *G* as the relative rate of volume *V* change, with Φ_eff_ the effective extensibility, ε_gemma_ the elastic deformation, and *y* the deformation threshold for growth to occur (*G* is set to 0 if ε_gemma_ < *y*).

We tried to interpret our mechanical data in terms of growth using Eq. 1. First, effective extensibility is predicted to be enhanced in *fer*-2 because it is softer than WT (Fig. 5C), which should lead to faster growth. Second, ε_gemma_ is rather similar between WT and *fer*-2 at 8 hours and is even higher for *fer*-2 at 1 hour, which also leads to faster growth. Both effects are in contrast with observations of slower growth in *fer*-2 than in WT (Fig. 1C). Therefore, mechanical parameters cannot explain differences in growth between *fer*-2 and WT.

Because those measurements are global and might hide local mechanical features relevant for growth, we turned to the measurement of mechanical parameters at a smaller (although supracellular) scale. We analyzed local deformations during the osmotic steps and interpreted them in terms of mechanical parameters to create average map of stiffness and turgor [Fig. 5, F and G, and detailed in Materials and Methods]. While turgor maps seem rather homogeneous (Fig. 5G), stiffness maps display a strong radial patterning, with the outer region being softer than the inner region (Fig. 5F). This radial patterning is much stronger in WT than in *fer*-2 (Fig. 5F). The local map of elastic deformation ε_gemma_ is also patterned in WT (especially at 8 hours), which is reminiscent of local growth rate patterns at the same time (Figs. 5H and 2D). Patterning of local elastic deformation is almost absent in *fer*-2 (Fig. 5H). Together, *FERONIA* regulates both stiffness and turgor of gemmae, which might be locally associated to growth pattern in each genotype but cannot explain global growth differences between *fer*-2 and WT.

### A mathematical model of the regulation of extensibility by FERONIA recapitulates growth phenotypes

Given that growth regulation by *FERONIA* does not arise from the regulation of the elastic deformation, we next considered the alternative hypothesis that *FERONIA* regulates extensibility. We took advantage of our extensive dataset and analyzed the relation between instantaneous growth rate averaged between 6 and 8 hours after imbibition and elastic deformation ε_gemma_ at 8 hours. Following Eq. 1, we fitted this relation to line, the slope of which corresponds to extensibility. We found that extensibility is higher in WT than in *fer*-2, although the spread of the data does not allow a strong conclusion (Fig. 6A).

To understand how *FERONIA* may control growth through the regulation of extensibility, we built a minimal mathematical model that predicts growth by accounting for the regulation of extensibility by integrity sensing (Fig. 6B). The model is based on the following considerations.

1) Growth rate *G* is proportional to deformation in excess of a threshold, as described by the Lockhart equation, modified with the contribution of the elastic modulus *E*_gemma_ (Eq.1) (*37*, *44*).

2) We hypothesized that the effective extensibility is set by the activity of a putative remodeling agent that represents all potential remodeling agents. Thus, extensibility is proportional to the concentration of this agent *W*, $\Phi_{\text{eff}}=\Phi_{w}W$, where Φ*_w_* is the agent molar extensibility; this decomposition was previously used to model pectin remodeling during pollen tube expansion (*45*). In the light of immunolocalization results on cell wall polysaccharides, variations in agent concentration *W* might be ascribed to variations of the concentrations of enzymes involved in the synthesis, the delivery, or the remodeling of matrix polysaccharides.

3) We assume that the remodeling agent is synthesized and integrated into the cell wall at a basal rate of $k_{w}^{+}$ and at a rate proportional to growth, with a proportionality factor β, as proposed in the context of protein synthesis during animal cell growth (*46*). The remodeling agent is degraded at a rate $k_{w}^{-}$ and diluted by wall expansion at rate *G*. Our core hypothesis is that *W* is affected by the activity of FERONIA and by signaling through FERONIA, which is described by a function S to be defined hereafter. The dynamics of agent concentration thus follows

$$\frac{dW}{dt}=k_{w}^{+}+\beta G-k_{w}^{-}W-\mathrm{GW}+S$$ (2)

4) Quantification of growth showed that FER affects both dynamics of growth (by speeding up germination) and equilibrium growth rate (by increasing it). We thus hypothesized that the effect of FERONIA/MARIS on extensibility is regulated by both a dynamical parameter, responsible for germination speed, and a static parameter, responsible for equilibrium growth rate. The minimal natural parameters are elastic strain (deformation $\varepsilon_{\text{gemma}}=P_{\text{gemma}}/E_{\text{gemma}}$, the static parameter) and irreversible strain rate (growth rate *G*, the dynamic parameter) (*47*). We thus write the sensing function S in the form

$$S=s\left(\varepsilon_{\text{gemma}}-t_{s}G\right)$$ (3)

where *s* is the strength of the feedback and *t*_s_ a characteristic time scale to sense elongation. The signs of the two terms are constrained by experimental observations. In *fer*-2 compared to WT, sensing is reduced (*s* is smaller), deformation ε is roughly equivalent, and extensibility is reduced (growth is slower), hence a positive sign for the first term. In *fer*-2 compared to WT, the increase in extensibility is slower (germination is retarded), hence a negative sign for the second term.

To sum up, the model predicts the growth rate of a plant assuming extensibility is modulated by integrity sensing. This modulation incorporates information from elastic deformation, which promotes extensibility, and from growth, which inhibits extensibility.

We used this model (Eqs. 1 to 3) to predict germination time and equilibrium growth rate, and we compared these predictions to experimental observations (Fig. 1, D to F, and fig. S4C). We first determined plausible ranges for the parameters based on literature values (table S1) and checked that predictions of *G*_eq_ and *T*_start_ are comparable in magnitude to their experimental values (model parameter exploration in fig. S4, D and E).

We then assessed consistency of model predictions with observations. Gemma volume is predicted to change slowly initially and then increase exponentially (Fig. 6C), which resembles experimental behavior (Fig. 1, C and F). Growth rate increases from 0 at the time corresponding to imbibition and reaches a plateau (Fig. 6C), which is also observed in experiments (fig. S4C), albeit with oscillations at later times. We analyzed the model and solved it numerically (“Supplementary text”). We confirmed that a decrease of sensing strength *s* reduces growth (decreases equilibrium growth rate *G*_eq_; Fig. 6D) and retards germination (decreases germination time τ_1/2_; Fig. 6E), as observed when mutating *FERONIA* (Fig. 1, D and E).

Together, the sensing of both elastic deformation and growth by FERONIA pathway is sufficient to explain gemmae growth phenotypes.

### The model predicts responses to osmotic pressure mediated by FERONIA

**Fig. 7.**
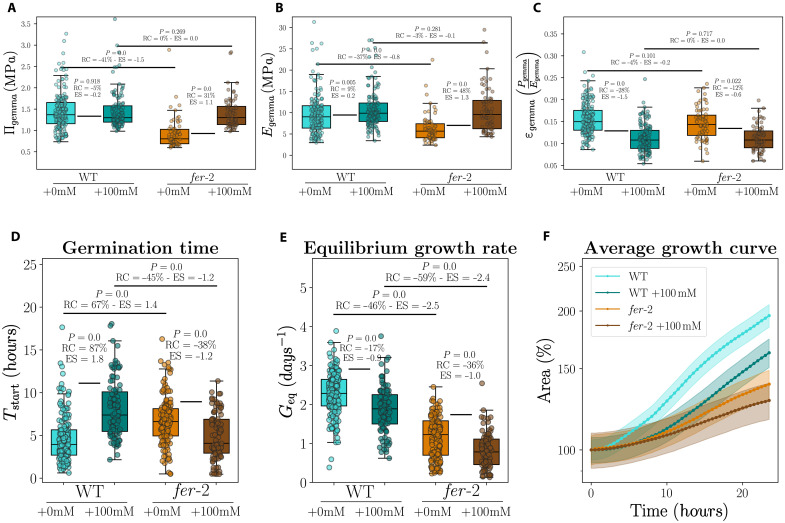
FERONIA responds to deformation and regulates growth. (**A** to **C**) Box plot and scatter plot of the mechanical properties for 8 hours after imbibition WT and *fer*-2. Plants were grown in reference medium with +0 mM or with +100 mM mannitol. (A) Osmotic pressure Π_gemma_, (B) volumetric elastic modulus *E*_gemma_, and (C) elastic deformation ε_gemma_ are plotted for each condition. WT(+0 mM): *n* = 193, rep. = 4. WT(+100 mM): *n* = 136, rep. = 3. *fer*-2 (+0 mM): *n* = 69, rep. = 6. *fer*-2 (+100 mM): *n* = 70, rep. = 3. (**D** to **F**) Parametrization of growth of WT as well as of *fer*-2 for plants grown in reference medium with +0 mM and with +100 mM mannitol. (**D**) Box plot and scatter plot of the germination starting time *T*_start_. (**E**) Box plot and scatter plot of the equilibrium growth rate *G*_eq_. (**F**) Average area over time (relative to initial area), the shaded areas correspond to the 95% confidence interval. WT: *n* = 178, rep. = 5. WT(+100 mM): *n* = 99, rep. = 3. *fer*-2: *n* = 141, rep. = 3. *fer*-2 (+100 mM): *n* = 94, rep. = 3.

We next considered mechanical perturbations to test our model. We varied the value of the elastic deformation ε_gemma_ in the model and examined changes in equilibrium growth rate and germination time (see below). Experimentally, we sought to affect elastic deformation using long-term, mild osmotic treatments by addition of 100 mM mannitol in the culture medium. Because gemmae might osmoregulate, i.e., increase their content in osmolytes to compensate for the external increase so as to maintain their turgor pressure, we first quantified osmotic pressure under permanent osmotic treatment. Osmotic pressure is increased in treated *fer*-2 (compared to untreated), indicating osmoregulation, while the WT shows no osmoregulation (Fig. 7A). To fully assess the mechanical state of gemmae, we also measured the elastic modulus. *fer*-2 appears stiffer under treatment, while WT shows a moderate increase in stiffness (Fig. 7B). The mechanical properties of WT gemmae are less affected by the treatment than *fer*-2 gemmae, consistent with the notion that FER is involved in mechanical responses. Last, we examined elastic deformation ε_gemma_ in these experiments. ε_gemma_ is diminished in similar proportions under treatment in WT and *fer*-2 (Fig. 7C).

We then compared model predictions for germination time and growth rate with observations. The equilibrium growth rate *G*_eq_ is predicted to decrease when elastic deformation is reduced, regardless of the value of the sensing parameter *s*, meaning that this behavior is expected for all genotypes (Eq. 8 and Fig. 7E). As predicted, if gemmae are continuously treated with +100 mM (Fig. 7F for experimental data), then the equilibrium growth rate *G*_eq_ decreases in WT as well as in *fer*-2 (Fig. 7E). In contrast, changes in germination time (quantified by τ_1/2_ in the model) according to elastic deformation are predicted to depend on the sensing parameter *s*. If *s* is high (typically 0.9 U hour^−1^, to mimic WT), germination is predicted to be delayed when elastic deformation is reduced, whereas if *s* is low (typically 0.05 U hour^−1^, to mimic *fer*-2), germination is predicted to occur earlier when elastic deformation is decreased (Eq. 9 and Fig. 6E). In agreement with predictions, germination is retarded under treatment in WT, while it is accelerated in *fer*-2 (Fig. 7D). Together, these results support the hypothesis that extensibility is positively regulated by elastic deformation and negatively regulated by growth rate through FERONIA.

### FERONIA regulates variability of growth

It was proposed that responses to mechanical signals provide feedback loops to ensure developmental robustness (*48*–*50*). Our mathematical model of the regulation of extensibility by FERONIA involves several feedback loops, suggesting a function in the regulation of growth variability. Mutation of *FERONIA* in *Arabidopsis* increases local growth variability in the root (*18*). We therefore investigated the regulation of growth variability of gemmae by *FERONIA*.

**Fig. 8.**
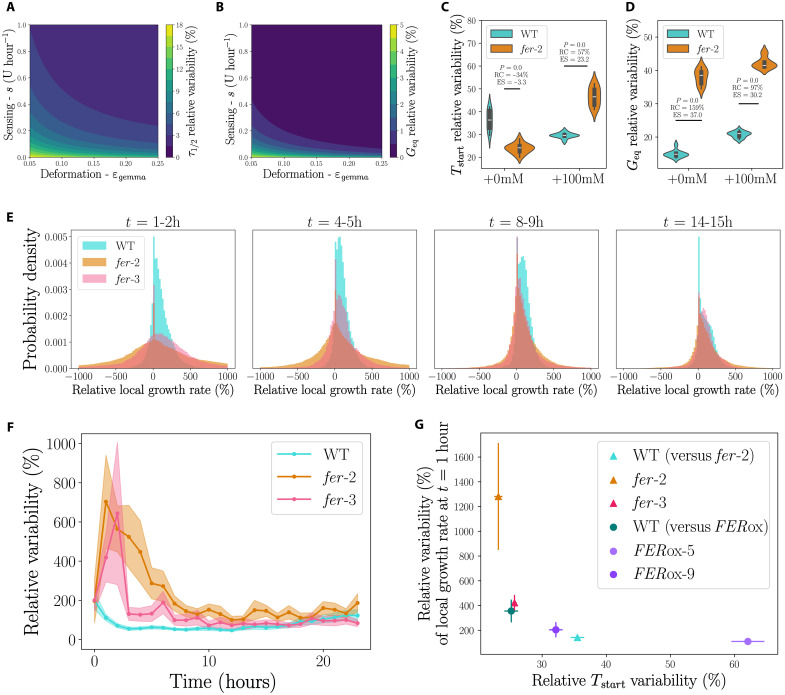
*FERONIA* regulates growth variability. (**A** and **B**) Relative variability of the growth parameters of the mathematical model according to the sensing parameter *s* and the elastic deformation ε_gemma_ for $k_{w}^{+}$ = 0.1 hour^−1^, $k_{w}^{-}=0.1\;{\text{hour}}^{-1}$, $t_{s}=1\;\text{hour}$, β=0.5 U, E=10 MPa, and Y=0.01 of the germination time τ_1/2_ (given by $|\frac{\tau_{1/2}\prime w_{0}}{\tau_{1/2}}|$) (A) and of the equilibrium growth rate *G*_eq_ (given by $\langle \frac{\Delta G^{2}}{G_{\text{eq}}^{2}}\rangle$) (B). (**C** and **D**) Violin plot of the relative variability in experiments (estimated by the AAD normalized by the mean) of *fer*-2 and WT at +0 and +100 mM for *T*_start_ (C) and *G*_eq_ (D). WT: *n* = 178, rep. = 5. WT(+100 mM): *n* = 99, rep. = 3. *fer*-2: *n* = 141, rep. = 3. *fer*-2 (+100 mM): *n* = 94, rep. = 3. (**E**) Probability density of local growth rate (normalized by its median) for WT, *fer*-2, and *fer*-3 at different times after imbibition. (**F**) Quantification of the variability (AAD over the median) of the local growth rate during 24 hours of growth, for WT, *fer*-2, and *fer*-3; the shaded areas represent the 95% confidence interval. WT: *n* = 61, rep. = 3. *fer*-2: *n* = 57, rep. = 3. *fer*-3: *n* = 50, rep. = 3. (**G**) Relative variability of the local growth rate at 1 hour (mean AAD over the median) as a function of the relative variability of *T*_start_ for *fer*-2, *fer*-3, *FER*ox-5, and *FER*ox-9, as well as associated WTs. Error bars are 90% confidence interval. WT (versus *fer*): *n* = 61, rep. = 3. *fer*-2: *n* = 57, rep. = 3. *fer*-3: *n* = 50, rep. = 3. WT (versus *FER*ox): *n* = 84 and rep. = 4. *FER*ox-5: *n* = 32 individuals, rep. = 3, and *FER*ox-9: *n* = 39 individuals and rep. = 3.

We first reexamined our mathematical model regarding variability. We estimated the variability of the germination time τ_1/2_ as its relative sensitivity to the initial value of the remodeling agent *W*; we defined the variability of the equilibrium growth rate *G*_eq_ as its relative standard deviation under noise in the production of the remodeling agent (supplementary model). The model predicts an increase of variability in germination characteristic time when sensing is decreased and, to a lesser extent, when elastic deformation ε_gemma_ is decreased (Fig. 8A). The same trends are predicted for the variability in equilibrium growth rate *G*_eq_ (Fig. 8B). These predictions are consistent with the idea that *FERONIA* promotes growth robustness through feedback loops.

We then tested these predictions and quantified experimental variability of the germination time *T*_start_ and equilibrium growth *G*_eq_ for WT plants and *fer* mutants under +0 and +100 mM osmotic conditions (Fig. 8, C and D, and fig. S6, A and B), as well as on overexpressor lines FERox under +0 mM osmotic condtion (fig. S6, C and D). Considering the equilibrium growth rate *G*_eq_, it is clear that a decrease of FERONIA activity induces a higher variability. Both *fer*-2 and *fer*-3 have a higher variability than WT, while *FER*ox-9 and *FER*ox-9 have a lower variability than WT (Fig. 8D and fig. S6, B and D). The variability of the equilibrium growth rate is also increased by a reduced elastic deformation (meaning under +100 mM osmotic treatment) in WT and *fer*-2 (Fig. 8D). All observations are in line with predictions, confirming the role of FERONIA, together with elastic deformation, in reducing the variability of the equilibrium growth rate *G*_eq_. Variability of the germination time *T*_start_ is also increased in the *fer*-2 mutant compared to WT, but only for gemmae growing under +100 mM osmotic stress (Fig. 8C). For the reference culture conditions (+0 mM), the variability of the germination time *T*_start_ seems to decrease in the *fer*-2 mutant (Fig. 8C and fig. S6A) and increases in *FER* overexpressor lines (fig. S6C). Experimental results disagree with predictions in normal culture medium and agree under osmotic treatment. To sum up, *FERONIA* promotes a decrease of variability of gemmae growth, with the notable exception of germination time under normal turgor pressure.

To understand why the variability of germination time is lower in *fer* mutants than in WT (and higher in *FER*ox lines) in the reference culture medium (+0 mM osmolytes), we considered spatial variations of the growth rate. We noticed a broader distribution of local growth rate (normalized by the mean) in *fer*-2 and *fer*-3 mutants, compared to WT (Fig. 8E). When plotting the relative width of growth distributions as a function of time (Fig. 8F), it appeared that there is an initial peak of variability before germination in the two *fer* mutants, while initial variability is lower in *FER*ox-9 and *FER*ox-9 compared to WT (fig. S6E). Two important remarks follow. First, at local scale, even in the reference medium (+0 mM osmolytes), mutating *FERONIA* increases variability. Second, there is a correlation between high local growth variability before germination and low global germination time *T*_start_ variability. This correlation is visible for the different mutants (Fig. 8G). One interpretation of this correlation is that, in the absence of FERONIA, there is a higher stochasticity of local growth rate that averages on the whole gemmae and leads to a more reproducible behavior, while, if FERONIA is present, this local stochasticity is diminished and the whole gemmae behavior is more coordinated. So, in WT, there is averaging over less variability, and the global behavior appears less robust, with more variations between individuals. Spatiotemporal averaging reduces organ scale variability, as shown by models developed for *Arabidopsis* sepals (*51*).

## DISCUSSION

We provided evidence that FERONIA regulates growth of *Marchantia* gemmae, in line with previous studies highlighting its role in growth of *Arabidopsis* and *Marchantia* (*9*–*13*, *16*, *19*, *52*, *53*). We showed that FERONIA positively regulates germination of gemmae. In *Arabidopsis* seeds, *FERONIA* was found to promote germination arrest in response to abscisic acid (*54*). Whereas abscisic acid was found to inhibit rhizoid emergence during germination of *Marchantia* gemma (*55*), germination of the whole gemma does not seem to be sensitive to abscisic acid (*37*), which makes it unlikely that the regulation of gemma germination by *FERONIA* depends on abscisic acid. For pollen tubes, it was postulated that the lack of pollen tube development in a double mutant for *FERONIA* homologs, *ANXUR1* and *2*, was rather due to spontaneous bursting just after emergence rather than a defect in germination (*56*). Nevertheless, during early gemmae growth, we did not observe cell death contrary to what is visible for *fer* mutants at later stages (*19*), which invalidates cell death as a potential explanation for *fer* early phenotype. We found that FERONIA promotes both cell proliferation and cell expansion and that FERONIA is necessary for the patterning of growth in gemmae. In At*fer* roots, proliferation defects can be observed (*57*), while the number of cells is not affected in seeds (*10*). So the regulation of proliferation by FERONIA seems to vary depending on organ context. Cell elongation was observed to be promoted by FERONIA in *Arabidopsis* seeds (*10*) and in *Marchantia* thallus (*19*), and defective growth patterns have been quantified (*18*). Therefore, we explored regulation of germination through the lens of the regulation of growth rather than the regulation of dormancy. Based on the observation that cell wall composition and/or structure was altered in *fer*-2, we challenged two hypotheses for the origin of the regulation of growth by FERONIA: regulation of properties related to tissue elastic deformation and regulation of cell wall extensibility.

Cell wall polysaccharides are good candidates for the regulation of both elasticity (*58*, *59*) and extensibility (*60*, *61*) by FERONIA. We screened families of cell wall polysaccharides by immunolabeling and demonstrated that FERONIA affects composition and/or remodeling of cell wall polysaccharides, and notably of xyloglucans. A recent transcriptomic analysis comparing *fer*-1 to WT highlights a potential regulation of xyloglucan remodeling by FERONIA (*62*). We may hypothesize a role for endotransglucosylase/hydrolase (XTH) family, which participates in xyloglucan remodeling. Xyloglucan:xyloglucosyl transferase activity is significantly enriched among the down-regulated transcripts in that study (the corresponding GO:0016762 is significantly enriched with statistical significance P≤0.001 in *fer*-1). Similarly, in At*fer* (*63*), down-regulated transcripts and proteins are enriched in the “cell wall–related genes,” notably, glycosyl hydrolase and, in particular, XTH (GO:0010411 for xyloglucan metabolic process is significantly enriched among the down-regulated transcripts with statistical significance *P* = 1.3 × 10^−6^). Remodeling of xyloglucan was indeed associated to regulation of extensibility or of cell wall elastic properties. Reduced xyloglucan labeling in xyloglucan backbone biosynthesis mutants was associated with decrease in cell wall stiffness and in turgor but no growth phenotype (*64*), while xyloglucan side-chain biosynthesis mutants *xxt1/xxt2* associate xyloglucan reduction to growth and morphogenesis phenotypes and extensibility reduction (*65*, *66*) but no significant effect on mechanical properties (*67*). Overexpression of *Arabidopsis* xyloglucan XTH leads to a stimulation of hypocotyl growth and extensibility as well as effects on cell wall elasticity and deposition (*68*). Therefore, xyloglucan remodeling may account for changes in cell wall mechanics in *fer*-2 and is a plausible explanation of extensibility changes, potentially leading to dual role of *FERONIA* in mechanics and growth.

Cellulose contents might be affected as well. Our immunolabeling results point at a limited modification of cellulose content or structure. Nevertheless, the transcriptomic analysis in (*62*) shows that expansins, cell wall proteins that remodel cellulose, are among the gene family significantly enriched in Mp*fer* down-regulated transcripts (P≤0.01).

Last, FERONIA affects composition and/or remodeling of some pectins (esterified homogalacturonan and rhamnogalacturonan-I). The transcriptomic analysis in (*62*) does not reveal a role of pectin remodeling or of pectin synthesis by *FERONIA*. Nevertheless, esterification of homogalacturonan is a well-characterized regulator of plant growth, organogenesis, and extensibility, as well as of cell mechanics (*69*). Rhamnogalacturonan-I is also thought to participate in the regulation of cell wall extensibility, although evidence is more scarce (*60*). Immunolabeling allows making assumptions on possible mechanisms mediating the regulation of elasticity and extensibility by FERONIA, directly in the cell wall. Nevertheless, identifying specific mechanisms for the regulation of extensibility requires the analysis of the presence and activity of remodeling proteins.

We demonstrated a significant effect of FERONIA on the mechanical properties of gemmae. FERONIA is required to maintain a radial pattern of elastic modulus, with softer gemma periphery. This is consistent with indentation-based measurements in *feronia* mutants showing either reduced stiffness or reduced turgor, in *Arabidopsis* (*21*) and in *Marchantia* (*19*), and with Brillouin microscopy-based measurements in At*theseus* mutant, another *Cr*RLK1 member (*35*). Here, we independently measured elastic modulus and turgor pressure, and we showed that FERONIA promotes build-up of both stiffness and turgor. In *Arabidopsis* roots, FERONIA controls the expansion of the vacuole (*12*), and so is potentially involved in turgor regulation.

We found that, in a given genotype, growth rate correlates with the elastic deformation ε_gemma_, as proposed in a chemorheological modeling framework for wall expansion (*45*) and partially tested in *Marchantia* (*37*). Nevertheless, elastic deformation is unaffected (or even increased) in *fer*-2 gemmae, which does not explain their slower growth compared to WT. Inspired by the proposal that strain-stiffening patterns growth domains (*70*), it is tempting to associate radial patterns of growth and of elastic modulus as both patterns are lost in *fer*-2. We may speculate that the slowly growing central region stiffens as a response to elastic deformation, reducing growth there.

We thus uncovered a dual role for FERONIA: It independently controls tissue properties governing elastic deformation (stiffness and turgor) and growth. This implies that FERONIA regulates extensibility, i.e., the ability of the cell wall to expand under a given tension.

**Fig. 9.**
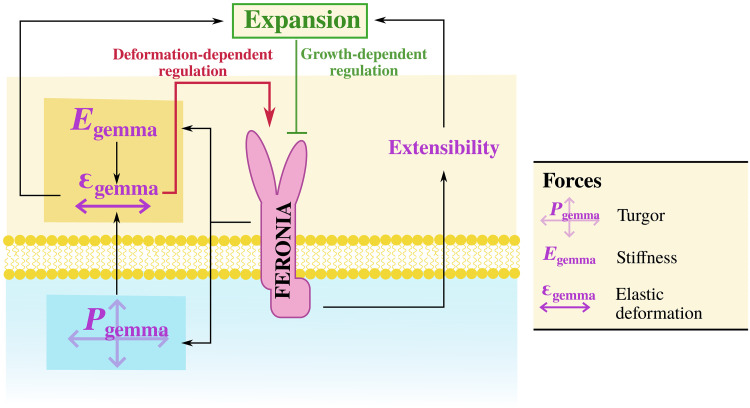
Molecular interpretation of the dual function of the FERONIA pathway. Dual role of FERONIA on mechanical parameters (turgor and stiffness) and on the extensibility. The twofold regulation of FERONIA by a positive deformation-dependent and a negative growth-dependent regulation is also represented.

We developed a mathematical model of the regulation of extensibility by FERONIA that recapitulates the main growth phenotypes of WT and *fer*-2. The model is based on the assumption of a double regulation of extensibility by a positive response to deformation and by a negative response to growth (Fig. 9). The assumption of a response to deformation is reinforced by the mechanical response of gemmae to a long-term osmotic treatment. The elastic modulus *E*_gemma_ and the osmotic pressure Π_gemma_ in *fer*-2 are lower than in WT under control growth condition but similar with +100 mM osmotic treatment, which corresponds to low deformation ε_gemma_. In *Arabidopsis*, FERONIA is inactivated under salt stress (*71*), suggesting an activation of FERONIA by deformation.

We now discuss the possible mechanistic basis of the double regulation of extensibility by FERONIA. We may first consider two distinct activation mechanisms upstream of FERONIA. The response to cell wall deformation could originate in the activation of FERONIA by the cell wall status, by binding to pectins either directly through the malectin domain (*21*, *22*) or indirectly through leucine-rich repeat extensins (*12*). We may then hypothesize that the response to cell wall expansion originates in RALF sensing (*23*). This would be possible if RALF synthesis depended on cell growth or if dilution of RALF by expansion were sensed by FERONIA, for instance. However, it is unclear whether pectin and RALF sensing can be uncoupled. It was hypothesized that RALF and pectin signals interact together through liquid-liquid phase separation based on formation of RALF-pectin condensate that act as stress sensor and promotes FERONIA clustering and massive endocytosis (*72*). Structural roles of some RALFs in the cell wall might also underlie a role of RALF as another mechanical sensor, and so RALF would rather mediate response to deformation (*17*). Therefore, we may second consider that the sensitivities to elastic deformation and to growth are properties of the signaling pathway downstream of FERONIA, which would filter static (deformation) and dynamic (expansion) signals. We can notice that rapid response of FERONIA is associated with growth arrest as apoplasmic pH increases, and cytosolic calcium or ROS increases, and can be directly involved in cell wall remodeling (*7*, *11*), while longer-term regulation through transcriptional regulation may be sensitive to cell expansion (*73*). This is well illustrated by RALF1-induced root growth arrest in *Arabidopsis*, which involves rapid (approximately minutes) alkanization of the apoplasm and slow (approximately hours) auxin synthesis (*17*, *74*).

Individual-to-individual (or organ-to-organ) variability differs in extent and in potential function according to context. Seed-to-seed variability in germination is relatively large, associated with a bet-hedging strategy to survive in an unpredictable environment (*75*), whereas sepal-to-sepal variability in morphology is relatively small, potentially associated with their function in flower protection (*51*). Here, we found that FERONIA is involved in limiting growth variability, like FERONIA in *Arabidopsis* roots (*18*). The regulatory network underlying our model comprises a FERONIA-dependent negative feedback on growth; such negative autoregulation is known to promote robustness to external perturbations (*76*). We found that, in reference culture conditions, FERONIA restricts variability in germination time, which we ascribed to the spatiotemporal averaging of gemma growth, similar to *Arabidopsis* sepals for which spatiotemporal averaging yields robust final shape (*51*). In the shoot apical meristem of *Arabidopsis*, it was also found that reducing the response to mechanical force, by reducing microtubule alignment, amplifies the variability of cell growth rate, which increases spatial averaging and so shape robustness (*50*). Together, we have provided a quantitative framework that we expect to be broadly applicable to the analysis of mechanical responses during development.

## MATERIALS AND METHODS

### Plant growth and mechanics: Experimental setup

#### Plant culture

Mother plants of the gemmae are grown at 22°C and 4600 to 5100 lx continuous light in a growth cabinet (Aralab) on solid Gamborg 1/2 (Gamborg B5 medium, Duchefa with 1.2% agar, Duchefa), with additional 1% sucrose (Sigma-Aldrich) for the *fer*-2 and *fer*-3 plants and the associated WT *M. polymorpha* subsp. *ruderalis* accession Takaragaike-1 (Tak-1 or WT), or without additional sucrose for the *FER*ox-5 and *FER*ox-9 plants and the associated WT. Details on the used lines are available in table S3, and the *FERONIA* gene ID is Mp4g15890 or Mapoly0869s0001. For the dividing nuclei staining, trypan blue staining, immunostaining, and microindentation assays, plants were first grown on plates in liquid Gamborg 1/2.

#### Culture and mechanical assays in a chip

Every growth and mechanical assay of this study was made by cultivating gemmae up to 24, 8, or 1 hour in a microfluidic chip. Chips and plants are prepared and grown as described in (*37*), except that the surfactant (Tween 20) was diluted at 1/2000 instead of 1/1000. For continuous osmotic treatments, we added 100 mM of d-mannitol (Sigma-Aldrich) to the Gamborg 1/2 liquid medium.

To measure mechanical parameters, gemmae were grown for 1 or 8 hours in a microfluidic chip and deformed by successive osmotic steps of +100, +250, and +900 mM of mannitol (Fig. 5A). Each step last 20 min, which includes 10-min medium change (at rate 25 μl/min) and 10 min of slower medium flow (at rate 8.33 μl/min) to reach mechanical equilibrium.

For gemmae area measurements, image acquisition was performed in bright field at 16× magnification using a Zeiss Axio Zoom V16, as in (*37*). For growth assays, images were recorded every 30 min, and for mechanical assays, images were recorded every 30 s; the acquisition started 2 min before the first osmotic step.

### Statistical analyses

Statistical significance of the comparison between two samples was estimated using a nonparametric Wilcoxon test (the *P* values are displayed and set to 0 when <10^−3^). To estimate the size effect of the difference between two samples, the relative change (RC; difference between the two median of the samples over the median of the reference sample) and the effect size (ES; median difference compared to the reference sample variability) were computed. For each experiment, *n* corresponds to the number of gemmae and rep. to the number of independent chip experiments or independent experimental replicates for experiments not conducted in chips.

Variability is estimated using the relative average absolute deviation (AAD). The relative AAD for a sample of median $\bar{x}$ is given by Eq. 4

$$\text{relative}\;\text{AAD}=\frac{1}{\bar{x}}\frac{1}{n}\sum_{i=1}^{n}|x_{i}-\bar{x}|$$ (4)

As samples present important size variation, the relative AAD for *T*_start_ and *G*_eq_ was computed on a random subset of values of size equal to 90% of the size of the smallest sample. The calculation was repeated on a number of random value subsets equal to the size of the subsets. For the local growth rate, a gemma AAD was computed for every individual over all local growth rate points. The median AAD over all individuals was then divided by median growth rate to avoid calculation error due to small local growth rate on individual gemmae. Correlation analysis was performed using a Spearman’s correlation coefficient calculation, and affine fits (for instantaneous growth rate *G*_instant_ correlation with ε_gemma_) are realized with the lineregress function from the scipy.stats library.

### Image analysis

All image analysis were performed using Python.

#### Area measurement

Image analysis to extract area evolution over time was performed as described in (*37*), although the size of the binary circular elements were adjusted for *fer*-2 as the image presented more irregularities (closing at 2.5 μm and opening at 20 μm).

#### Growth parameters computation

The two phases of growth are fitted by

$$\begin{array}{cc} A\left(t<T_{\text{start}}\right) & =A_{0}\\ A\left(t>T_{\text{start}}\right) & =A_{0}e^{G_{\text{eq}}\left(t-T_{\text{start}}\right)} \end{array}$$ (5)

with *A* being gemma area. *T*_start_ and *G*_eq_ are obtained from successive fits as described in (*37*). For the correlation analysis, we used the instantaneous growth rate $G_{\text{instant}}=\frac{1}{A}\frac{dA}{dt}$, computed as in (*37*).

#### Mechanical parameters computation

We aimed at determining the average internal osmotic pressure of a gemmae Π_gemma_ as well as the bulk volumetric elastic modulus *E*_gemma_ defined by Eq. 6

$$P_{\text{gemma}}=E_{\text{gemma}}\frac{V-V_{p}}{V_{p}}$$ (6)

with *V* being the gemmae volume, *V*_p_ the plasmolysis volume, and *P*_gemma_ the turgor pressure.

Gemmae are submitted to four osmotic concentrations: +0, +100, +250, and + 900 mM. Plants remain turgid under the three first concentrations and are plasmolyzed under 900 mM (Fig. 5A). Mechanical parameters were deduced from the water potential Ψ equilibrium between the outside medium and the gemmae internal medium at each turgid pressure step

$$\begin{array}{c} \Psi_{\text{gemma},\;0\;\text{mM}}=\Psi_{\text{out},\;0\;\text{mM}}\\ \Psi_{\text{gemma},\;100\;\text{mM}}=\Psi_{\text{out},\;100\;\text{mM}}\\ \Psi_{\text{gemma},\;250\;\text{mM}}=\Psi_{\text{out},\;250\;\text{mM}} \end{array}$$ (7)

with the different component of the water potential: the hydrostatic pressure (the turgor pressure *P*_gemma_ for the internal medium and no overpressure for the outside media) and the osmotic pressure Π

$$\begin{array}{c} \Psi_{\text{gemma}}=P_{\text{gemma}}-\Pi_{\text{gemma}}=E_{\text{gemma}}\frac{V-V_{p}}{V_{p}}-\Pi_{\text{gemma}}\\ \Psi_{\text{out}}=-\Pi_{\text{out}} \end{array}$$ (8)

with *V*_p_ being the plasmolysis volume given by the +900 mM step. This yields

$$\begin{array}{cc} E_{\text{gemma}}\frac{V_{0\text{mM}}-V_{p}}{V_{p}}-\Pi_{\text{gemma}} & =-\Pi_{\text{out},\;0\text{mM}}\\ E_{\text{gemma}}\frac{V_{100\text{mM}}-V_{p}}{V_{p}}-\Pi_{\text{gemma}}\frac{V_{0\text{mM}}}{V_{100\text{mM}}} & =-\Pi_{\text{out},\;100\text{mM}}\\ E_{\text{gemma}}\frac{V_{250\text{mM}}-V_{p}}{V_{p}}-\Pi_{\text{gemma}}\frac{V_{0\text{mM}}}{V_{250\text{mM}}} & =-\Pi_{\text{out},\;250\text{mM}} \end{array}$$ (9)

where $\frac{V_{0\;\text{mM}}}{V_{100/250\;\text{mM}}}$ accounts for the dilution effects at each deformation step, as we hypothesized that osmoregulation does not occur on these time scales and therefore the number of osmolytes is constant.

Two equilibria are needed to deduce both the elastic modulus *E*_gemma_ and the osmotic pressure Π_gemma_. As the gemmae exhibit a strain-stiffening behavior, we estimate Π_gemma_ using the equilibrium closer to the plasmolysis (+100 and + 250 mM) to stay in a similar strain regime

$$\begin{array}{c} \Pi_{\text{gemma}}=\\ \frac{V_{250\text{mM}}\Pi_{\text{out},\;250\text{mM}}}{V_{0\text{mM}}}\frac{V_{100\text{mM}}\left(V_{100\text{mM}}-V_{p}\right)}{V_{100\text{mM}}\left(V_{100\text{mM}}-V_{p}\right)-V_{250\text{mM}}\left(V_{250\text{mM}}-V_{p}\right)}\\ -\frac{V_{100\text{mM}}\Pi_{\text{out},\;100\text{mM}}}{V_{0\text{mM}}}\frac{V_{250\text{mM}}\left(V_{250\text{mM}}-V_{p}\right)}{V_{100\text{mM}}\left(V_{100\text{mM}}-V_{p}\right)-V_{250\text{mM}}\left(V_{250\text{mM}}-V_{p}\right)} \end{array}$$ (10)

The turgor pressure *P*_gemma_ before the shocks is calculated according to

$$P_{\text{gemma}}=\Pi_{\text{gemma}}-\Pi_{\text{out},\;0\;\text{mM}}$$ (11)

Last, *E*_gemma_ is estimated at the equilibrium corresponding to the initial medium

$$E_{\text{gemma}}=\frac{V_{p}}{V_{0}-V_{p}}\left(\Pi_{\text{gemma}}-\Pi_{\text{out},\;0\;\text{mM}}\right)$$ (12)

#### Measurement of local deformations

Local growth rate and local mechanical properties are assessed by first extracting local deformations on time courses of gemmae bright-field images. Several processing steps are performed.

1) Only gemmae with two notches and the attachment point visible are selected. For the osmotic step strain rate calculation, a time point in each equilibrium state is selected manually.

2) The localization of these three points (two notches and the attachment point) is detected manually for the first time point and is propagated to the next time points using contour fitting to adjust for movement and curvature to identify the notches (fig. S7A, step 1). Details of the computation: After binarization of the image, the gemma contour is detected using the openCV findContours function. High curvature points of this contour are calculated by fitting circles to contour portion, and the highest one is extracted using the find_peaks function of the scipy.signal library. Contours are aligned to the previous time point by minimizing the distance between the two contours, and the closest high sign peaks are associated.

3) Localization of the points of interest are used to align gemmae according to the following procedure: The image is rotated to have the notch-to-notch axis horizontal and translated so that the attached point stays at the same coordinate over time. These operations are performed using the openCV rotate and warpAffine functions (fig. S7A, step 1).

4) The optical flow, which is the apparent movement of the image objects, is computed on the processed image using the calcOpticalFlowFarneback function (openCV function applied to the image converted to grayscale with the following parameters pyr_scale = 0.5, levels = 3, winsize = 15, iterations = 3, poly_n = 5, poly_sigma = 1.1, and flags = cv.OPTFLOW_FARNEBACK_GAUSSIAN), which returns the *V_x_* and *V_y_* component of the velocity field (fig. S7A, step 2).

5) Background is removed using the same segmentation pathway as for area measurements.

6) To avoid noise caused by small movements on the image and to be able to detect tissue scale movement, the velocity field components are filtered using the ndimage.gaussian_filter of the scipy library with σ = 15 μm (fig. S7A, step 3).

7) Local deformations are calculated as being the trace of the gradient, i.e., $\frac{dV_{x}}{dx}+\frac{dV_{y}}{dy}$ (fig. S7A, step 4).

To assess the reliability of the method, the global growth rate calculated based on area ($\frac{1}{A}\frac{dA}{dt}$) was compared to the sum of the local deformation or growth rate for each time step (fig. S7B). Overall, the local growth rate computed with optical flow is correlated with the global one, although it tends to be reduced. Moreover, the dynamics over time look similar although noisier (fig. S7C).

#### Local growth rate quantification

To compare local growth rate between gemmae, images were co-registered by aligning the notch-to-notch axis (using the warpAffine function) and were scaled to the same area (using the openCV resize method). To visualize average patterns, median values of the local growth rate at a given localization are plotted only for data point with a gemmae presence probability higher than 0.5. Local probability of gemmae presence was computed by assigning a probability of $1/N_{\text{gemmae}}$ to each aligned gemma and thresholding the obtained map to 0.5.

To quantify spatial patterning of the local growth rate, we proceeded first to angular averaging. Each local growth rate field of a gemma (we work with resized field) is virtually dissected into 13 annuli centered at the mid-distance between the two notches and with increased radius covering the whole gemmae once (see schematic in Fig. 2E). The average local growth rate values was computed for each annulus.

For radial averaging, local growth rate field is virtually dissected into 36 angular sectors (centered at the middle of the two notches; see schematic in Fig. 2F). The average local growth rate value is computed for each sector.

#### Local mechanical parameters computation

Local displacement fields were also calculated at each equilibrium state of a given gemma. Gemmae were aligned, and median local deformation was calculated. Due to boundary effect on mechanical parameter evaluation, mechanical parameters are evaluated only on the surface with a probability of gemmae presence higher than 0.7. A similar reasoning than for the global mechanical parameters is applied for local elastic modulus and pressures. If we do not account for the dilution effect and we note the deformation from the equilibrium *i* to *j* $\varepsilon_{i\to j}={\frac{dU_{x}}{dx}}_{i\to j}+{\frac{dU_{y}}{dy}}_{i\to j}$, the local equilibrium equations are

$$\begin{array}{c} E_{\text{gemma}}\;\varepsilon_{\text{plasmo}\to 0\;\text{mM}}-\Pi_{\text{gemma}}=-\Pi_{\text{out},\;0\;\text{mM}}\\ E_{\text{gemma}}\;\varepsilon_{\text{plasmo}\to 100\;\text{mM}}-\Pi_{\text{gemma}}=-\Pi_{\text{out},\;100\;\text{mM}}\\ E_{\text{gemma}}\;\varepsilon_{\text{plasmo}\to 250\;\text{mM}}-\Pi_{\text{gemma}}=-\Pi_{\text{out},\;250\;\text{mM}} \end{array}$$ (13)

So we obtained the local Π_gemma_ as

$$\Pi_{\text{gemma}}=\frac{\Pi_{\text{out},\;250\;\text{mM}}\;\varepsilon_{\text{plasmo}\to 100\;\text{mM}}-\Pi_{\text{out},\;100\;\text{mM}}\;\varepsilon_{\text{plasmo}\to 250\;\text{mM}}}{\varepsilon_{\text{plasmo}\to 100\;\text{mM}}-\varepsilon_{\text{plasmo}\to 250\;\text{mM}}}$$ (14)

and so the local *E*_gemma_ is

$$E_{\text{gemma}}=\frac{\Pi_{\text{gemma}}-\Pi_{\text{out},\;0\;\text{mM}}}{\varepsilon_{\text{plasmo}\to 0\;\text{mM}}}$$ (15)

### Mechanical measurements with microindentation

Gemmae were grown in liquid medium for 1 or 8 hours and placed on double-sided adhesive tape within a petri dish. Gemmae were covered with a liquid solution of Gamborg 1/2 with 900 mM of mannitol. Measurements started 30 min after gemmae were placed in this high osmotic medium, so that gemma cells were fully plasmolyzed. Microindentation was performed using a TI 950 Triboindenter (Hysitron) with a flat end tip of radius *R* = 50 μm. Indentation was performed twice at each location to ensure that gemma was properly flattened on the petri dish and only the second measurement was taken into account. For each gemmae, measurements were done in the central part and in the growing periphery. The “displacement-control” mode was used (the imposed displacement is represented in fig. S3C). As automatic detection of the surface was not always efficient on softer gemmae, the imposed displacement consisted of a first negative ramp of 15 μm to be sure to be out of contact with the sample. Elasticity of the gemma was measured using the portion of the curve that corresponds to a positive indentation of 2500 nm with respect to the gemma surface at a pace of 1 μm/s (10 s retract, 15 s extend). Curves were recorded with 200 points/s (example of a load response to the imposed displacement in fig. S3D). The surface was detected as the point where the load starts to increase during the extend. It was automatically detected by filtering load oscillation using the savgol_filter function (scipy.signal, with a window length of 11 points and a polynomial of order 3) and setting a difference threshold of 0.02 μN to the filtered signal (so around 0.01% of the maximum measured load). From the calculated surface, 2.5 μm of indentation depth was used to calculate the indentation modulus, which was extracted as the slope of the load (*F*) increase with respect to the indentation depth (*d*) (example of a fitted portion of the curve in fig. S3E). Curves for which the surface was not detected were discarded from the analysis (less than 10% of the curves). The slope between the load and the deformation increase was converted into an indentation modulus *E*_indentation_ using the contact law between a rigid cylinder with flat end (the indenter) and an elastic half-space (the gemma)

$$F=2{\mathrm{RE}}_{\text{indentation}}d$$ (16)

### Microscopy and staining assays

#### Nuclei staining

Gemmae were collected from the mother plants and grown on liquid Gamborg 1/2 (Duchefa), in cell filters, in the growth cabinet for 0, 4, 8, or 19 hours. Gemmae in the cell filters were then soaked on Gamborg 1/2 with 10 μm EdU solution (from the Click-iT EdU Imaging Kits Protocol, Thermo Fisher Scientific) for 2 hours, 22°C under continuous light. The cell fixation, permeabilization, and EdU detection were performed as described in (*19*) (using the Click-iT kit with Alexa Fluor 647 dye). An additional phosphate-buffered saline (PBS) wash step (four times) was added both at the end of the permeabilization and detection phase. After soaking in chloral hydrate for 30 min, gemmae were washed four times in PBS and mounted in Immersol W 2010 oil solution (Zeiss) for imaging.

Imaging was performed using an inverted confocal microscope TCS SP8 (Leica) with the HC PL APO 20×/0.75 IMM CORR CS2 objective. The white light laser (Leica) was set to 647 nm for excitation (100%) and the HyD1 detector detection windows to 657 to 680 nm.

The number of nuclei was automatically detected using the ImageJ 3D Nuclei Segmentation plugin with the “MaxEntropy” algorithm for the automatic thresholding. The obtained segmented image was then projected using ImageJ. Nuclei number was assessed with the Analyze Particles tools by selecting big and round enough particles (size set over 8 μm^2^ and circularity over 0.2). Analysis was automated using the following macro

run(“Set Measurements...”, “area shape redirect=None decimal=3”);

open(“˜/example.tif”);

selectImage(“example.tif”);

run(“3D Nuclei Segmentation”, “auto_threshold=MaxEntropy manual=0

separate_nuclei”);

selectImage(“merge”);

run(“Z Project...”, “projection=[Max Intensity]”);

setAutoThreshold(“Default dark no-reset”);

setThreshold(1, 65535, “raw”);

setOption(“BlackBackground”, true);

run(“Convert to Mask”);

run(“Analyze Particles...”, “size=8-Infinity circularity=0.2-1.00

display clear add composite”);

selectImage(“merge”);

saveAs(“Tiff”, “˜/results/merge.tif”);

#### Cell death staining

To visualize dead cells, a trypan blue test was performed as described in (*19*), with an incubation time in trypan blue dye of 3 min to ensure proper staining of the gemmae and a decolorizing step of 30 min in choral hydrate. Prior to staining, gemmae were collected from the mother plants and grown on liquid Gamborg 1/2 (Duchefa) for 1 or 8 hours. For the 1-week *fer*-2 positive control, plantlets grew on solid Gamborg 1/2 for a week. Image acquisition was performed in bright field at 63× magnification (or 32× for WT 1-week-old plantlets) using a Zeiss Axio Zoom V16.

### Cell wall composition analysis

#### Resin embedding, immunofluorescence labeling, and staining

*Marchantia* gemmae were collected, and each condition (WT and *fer*-2) were processed with manual classical embedding adapted from (*77*) as follows. Samples were fixed for 1 hour and 30 min in 1% paraformaldehyde and 1% glutaraldehyde mixture (v/v) in 0.1 M sodium cacodylate buffer (pH 7.2) and washed (5 min) in ultrapure water. Then, the samples were dehydrated in an ethanol series (30, 50, 70, and 100%) for 2 hours each and embedded in LR White (LRW) resin through an LRW-ethanol series (25, 50, 75, and 100%) for 24 hours each. Last, they were embedded (24 hours) in LRW resin complemented with the ultraviolet (UV) catalyst benzoin methyl ether (0.5%, w/v) and polymerized for 48 hours at 4°C with UV light. Sections from resin blocks (2 μm; EM UC6 Leica microsystems) were collected on 10-well slides previously coated with poly-l-lysine (0.01%, v/v). For immunofluorescence labeling, the resin sections were blocked in PBS-Tween 20 0.1% (w/v) supplemented with 3% of bovine serum albumin (BSA) and normal goat serum (NGS; 1/20, v/v) for 30 min. Sections were washed in PBS-T + 1% BSA (5 × 5 min) and incubated overnight at 4°C in a wet chamber with the primary antibody (table S2). After washing in PBS-T + 1% BSA (5 × 5 min), the sections were incubated for 2 hours at 25°C in a wet chamber with a rat secondary antibody coupled to Alexa Fluor 647 (d:1/200, Invitrogen). Last, they were washed in PBS-T + 1% BSA (5 × 5 min) and ultrapure water (2 × 5 min). For histochemical staining of cellulose, the resin sections were incubated with the DR23 (1%, w/v) for 30 min in darkness. The resin sections were washed (3 × 5 min) with ultrapure water. Fluorescence on sections was observed using a macroscope Zeiss Axio Zoom with a fluorescence filter (Alexa Fluor 647; set 50, BP 640/30 BP690/50, DR23; mRF12, BP 572/25 BP 629/62), an exposure time of 400 ms, and a range of 50 μm, slides: 11, interval: micrometers. Negative controls were performed by the omission of the primary antibody or of probe DR23.

#### Image analysis and statistical test

Fluorescence intensity measurements were performed on a region of interest (ROI) containing the central area of gemma and used to obtain a mean of fluorescence intensity value of the ROI. To automate the measurements, an ImageJ macro was used, which was adapted from (*78*). It manages the opening of an image, the projection of average intensity along the *z* axis, and the retrieval and saving of intensities along a line in a result table. *Z*-average projection is used to smooth out cutting effects, as each point on the line is itself an average of a user-defined orthogonal segment.

## Supplementary Materials

### This PDF file includes:

Supplementary Text Figs. S1 to S7 Tables S1 to S3 References
