# Supplementary material for: Dual role of the receptor kinase FERONIA in regulating tissue mechanics and growth
Source: Sci Adv. 2026 Jul 15;12(29):eaeb8608. doi: 10.1126/sciadv.aeb8608 (PMC13371910; doi:10.1126/sciadv.aeb8608)
Supplement: Supplementary file 1 — Supplementary Text Figs. S1 to S7 Tables S1 to S3 References [file sciadv.aeb8608_sm.pdf]

Science Advances
AAAS

Supplementary Materials for

Dual role of the receptor kinase FERONIA in regulating tissue mechanics and growth

Elise Muller *et al.*

Corresponding author: Stéphanie Drevensek, stephanie.drevensek@polytechnique.edu;
Arezki Boudaoud, arezki.boudaoud@polytechnique.edu

Sci. Adv. **12**, eaeb8608 (2026)
DOI: 10.1126/sciadv.aeb8608

This PDF file includes:

Supplementary Text
Figs. S1 to S7
Tables S1 to S3
References

Supplementary Text

A simple estimate for the germination characteristic time τ.

By neglecting the dilution effect, which is reasonable to get an estimate of early growth dynamics, we get the following dynamics for W (equation S1),

$$\frac{\mathrm{d}W}{\mathrm{d}t} = k_w^+ + \beta G - k_w^- W + s\left(\varepsilon_{gemma} - t_s G\right). \tag{S1}$$

Thus G is given by:

$$\begin{aligned} G(t) &= \Phi_w(\varepsilon_{gemma} - y)_+((W_0 - W_{eq})e^{-t/\tau} + W_{eq}) \\ W_{eq} &= \frac{k_w^+ + s\varepsilon_{gemma}}{k_w^- + (st_s - \beta)\Phi_w(\varepsilon_{gemma} - y)_+} \\ 1/\tau &= k_w^- + (st_s - \beta)\Phi_w(\varepsilon_{gemma} - y)_+ \end{aligned} \tag{S2}$$

Solution of the complete model for W.

The complete model equation is given by

$$\frac{\mathrm{d}W}{\mathrm{d}t} = k_w^+ + \beta G - k_w^- W - GW + s(\varepsilon_{gemma} - t_s G). \tag{S3}$$

This can be solved analytically, as the right member of the equation can be re-organised as a second degree polynomial

$$\frac{\mathrm{d}W}{\mathrm{d}t} = -\Phi_w(\varepsilon_{gemma} - y)_+ W^2 - W(k_w^- + (st_s - \beta)\Phi_w(\varepsilon_{gemma} - y)_+) + k_w^+ + s\varepsilon_{gemma}. \tag{S4}$$

The roots of the polynomial, w_+ and w_-, are expressed are

$$\begin{aligned} w_\pm = & \frac{-k_w^- - (st_s - \beta)\Phi_w(\varepsilon_{gemma} - y)_+}{2\Phi_w(\varepsilon_{gemma} - y)_+} \\ & \pm \frac{\sqrt{(k_w^- + (st_s - \beta)\Phi_w(\varepsilon_{gemma} - y)_+)^2 + 4\Phi_w(\varepsilon_{gemma} - y)_+(k_w^+ + s\varepsilon_{gemma})}}{2\Phi_w(\varepsilon_{gemma} - y)_+}. \end{aligned} \tag{S5}$$

Equation S3 is thus equivalent to

$$\frac{\mathrm{d}W}{-\Phi_w(\varepsilon_{gemma} - y)_+(w_+ - w_-)}\left(\frac{1}{W - w_+} - \frac{1}{W - w_-}\right) = \mathrm{d}t. \tag{S6}$$

Integration of equation S6 gives the following expression for W (equation S7),

$$\begin{aligned} W(t) &= \frac{w_+ - \epsilon w_-}{1-\epsilon} \\ with\ \epsilon &= \frac{w_0 - w_+}{w_0 - w_-} e^{-\Phi_w(\varepsilon_{gemma}-y)_+(w_+-w_-)t} \\ and\ w_0 &= W(t=0). \end{aligned} \tag{S7}$$

The equilibrium value for the elongation agent surface concentration is $W_{eq} = w_+$. The equilibrium growth rate is thus given by

$$\begin{aligned} G_{eq} &= \Phi_w(\varepsilon_{gemma} - y)_+ w_+ \\ &= -\frac{1}{2}k_w^- - \frac{1}{2}(st_s - \beta)\Phi_w(\varepsilon_{gemma} - y)_+ \\ &+ \frac{1}{2}\sqrt{(k_w^- + (st_s - \beta)\Phi_w(\varepsilon_{gemma} - y)_+)^2 + 4\Phi_w(\varepsilon_{gemma} - y)_+(k_w^+ + s\varepsilon_{gemma})}. \end{aligned} \tag{S8}$$

To characterise germination, we define a germination half-time $\tau_{1/2}$ so that $W(\tau_{1/2}) = \frac{W_{eq}}{2}$, which gives

$$\tau_{1/2} = \frac{1}{\Phi_w(\varepsilon_{gemma} - y)_+(w_+ - w_-)} \ln\left(\frac{(2w_- - w_+)(w_0 - w_+)}{w_+(w_0 - w_-)}\right). \tag{S9}$$

Estimates of the parameter values are given in Table S1.

Variability estimates given by the model

We consider small fluctuations of the remodelling agent concentration W around an equilibrium value W_{eq}, which is solution of Equation (S1), so that $W = W_{eq} + \Delta W$ and $\Delta W \ll W_{eq}$, we linearise Equation S3 and get

$$\frac{\mathrm{d}\Delta W}{\mathrm{d}t} = -\mathcal{R}_{fluc}\Delta W + \eta(t), \tag{S10}$$

where

$$\begin{aligned} \mathcal{R}_{fluc} &= -(-2\phi(\varepsilon_{gemma} - y)W_{eq} + (\beta - s)\phi(\varepsilon_{gemma} - y) - k_w^-) \\ &= \sqrt{(k_w^- + (s-\beta)\phi(\varepsilon_{gemma} - y))^2 + 4\phi(\varepsilon_{gemma} - y)(k_w^+ + s\varepsilon_{gemma})}, \end{aligned} \tag{S11}$$

and η is a Gaussian white noise of amplitude D_η.

We therefore predict

$$\langle \Delta W^2 \rangle = \frac{D_\eta}{2\mathcal{R}_{fluc}}. \tag{S12}$$

This gives the following relative fluctuation amplitude for G_{eq}

$$\frac{\langle \Delta G^2 \rangle}{G_{eq}^2} = \frac{\langle \Delta W^2 \rangle}{W_{eq}^2} = \frac{D_\eta}{2W_{eq}^2 \mathscr{R}_{fluc}}. \tag{S13}$$

To evaluate the germination half-time fluctuation, we are interested in the sensitivity of the germination time to the initial value of W (w_0), which is defined by

$$\frac{\mathrm{d}\tau_{1/2}}{\tau_{1/2}} = \frac{\tau'_{1/2} w_0}{\tau_{1/2}} \frac{\mathrm{d}w_0}{w_0}. \tag{S14}$$

So the sensitivity of the relative germination time to the initial value of W is obtained from Equation S9 as

$$\frac{\tau'_{1/2} w_0}{\tau_{1/2}} = \frac{(w_+ - w_-) w_0}{(w_0 - w_+)(w_0 - w_-)} \frac{1}{\ln\left(\frac{(2w_- - w_+)(w_0 - w_+)}{w_+(w_0 - w_-)}\right)}. \tag{S15}$$

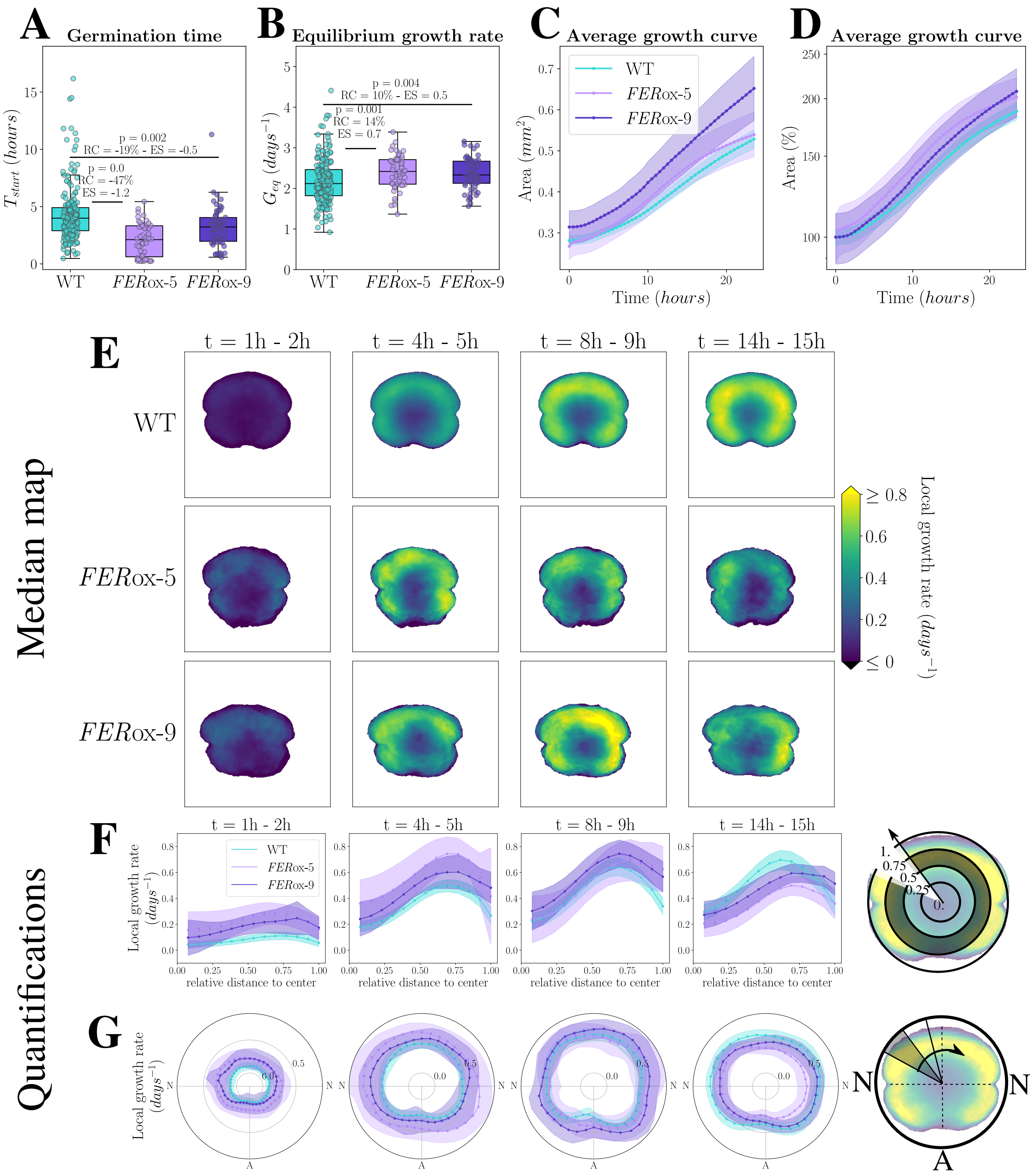

A
Germination time
T_{start} (hours)
p = 0.002
RC = -19% - ES = -0.5
p = 0.0
RC = -47%
ES = -1.2
WT
FERox-5
FERox-9
B
Equilibrium growth rate
G_{eq} ($days^{-1}$)
p = 0.004
RC = 10% - ES = 0.5
p = 0.001
RC = 14%
ES = 0.7
C
Average growth curve
Area (mm^2)
Time (hours)
D
Average growth curve
Area (%)
E
t = 1h - 2h
t = 4h - 5h
t = 8h - 9h
t = 14h - 15h
Median map
Local growth rate ($days^{-1}$)
≥ 0.8
≤ 0
F
Quantifications
relative distance to center
G
N
A

Figure S1: **Growth phenotypes of *FER*ox lines support the role of FERONIA in regulating growth rate and patterning.** (**A-D**) Parametrisation of growth of WT and of *FERONIA* overexpression lines *FER*ox-5 and *FER*ox-9. (**A**) Box plot and scatter plot of the germination starting time T_{start}. (**B**) Box plot and scatter plot of the equilibrium growth rate G_{eq}. (**C**) Average absolute area versus time. (**D**) Average area versus time (relative to initial area). Shaded areas are the 95% confident interval. WT: n = 190 individuals, rep. = 6. *FER*ox-5: n = 52 individuals, rep. = 3. *FER*ox-9: n = 57 individuals and rep. = 3. (**E**) Median local growth rate maps for WT and for *FER*ox-5 and *FER*ox-9. The median local growth rate is calculated over the individuals and over one hour at different time intervals (1 h-2 h, 4 h-5 h, 8 h-9 h and 14 h-15 h after imbibition). It is represented with a symmetric logarithmic color scale. WT: n = 84 and rep. = 4. *FER*ox-5: n = 32 individuals, rep. = 3. *FER*ox-9: n = 39 individuals and rep. = 3. (**F-G**) Radial and circumferential quantification of the local growth rate for WT, *FER*ox-5 and *FER*ox-9. (**F**) Mean local growth rate of the gemma surface at a given distance from the centre of the gemmae, averaged over all orientations. (**G**) Mean local growth rate of the gemma surface in a given angular sector, averaged over all distances to the centre. Shaded areas are the 95% confident interval. WT: n = 84 and rep. = 4. *FER*ox-5: n = 32 individuals, rep. = 3. *FER*ox-9: n = 39 individuals and rep. = 3.

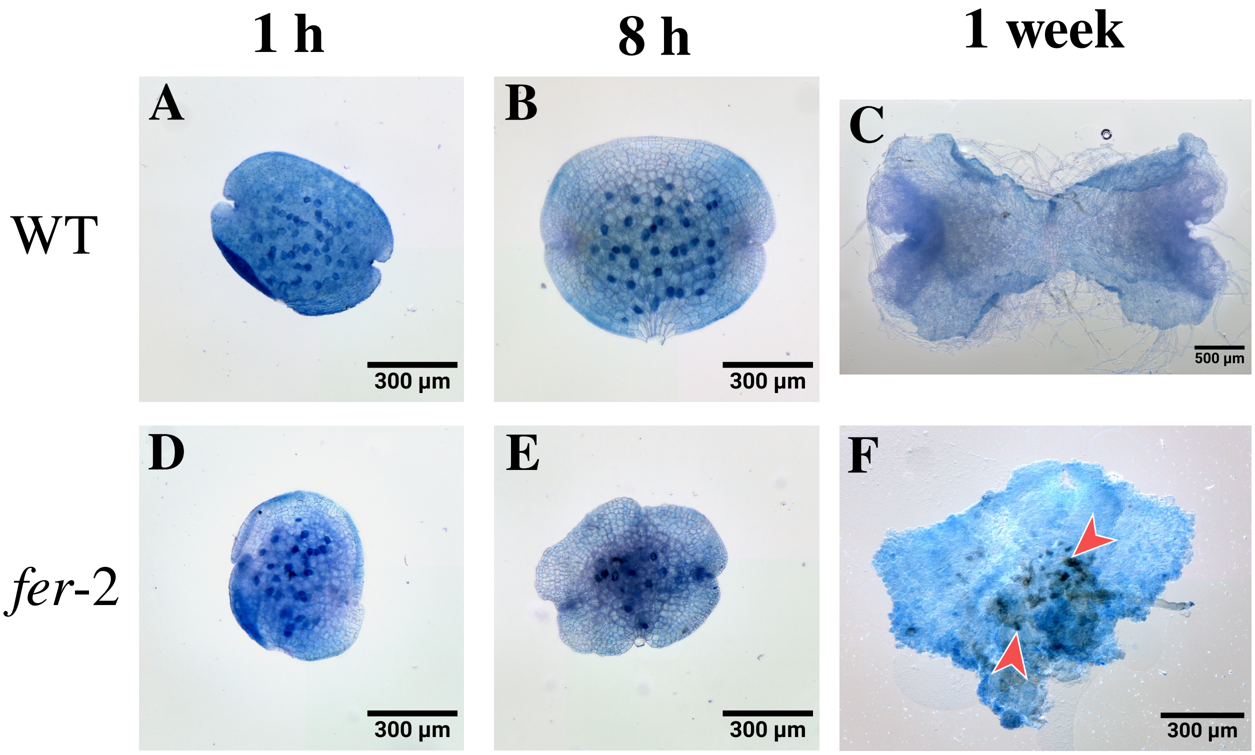

Figure S2: **WT and *fer*-2 do not present cell death during gemmae early growth.** Trypan blue staining of gemmae of WT at 1 h (**A**), 8 h (**B**) and 1 week (**C**), and of *fer*-2 at 1 h (**D**), 8 h (**E**) and 1 week (**F**) after imbibition. Images are representative gemmae from 15 individuals in each condition. Red arrow heads are pointing at examples of dead cell staining.

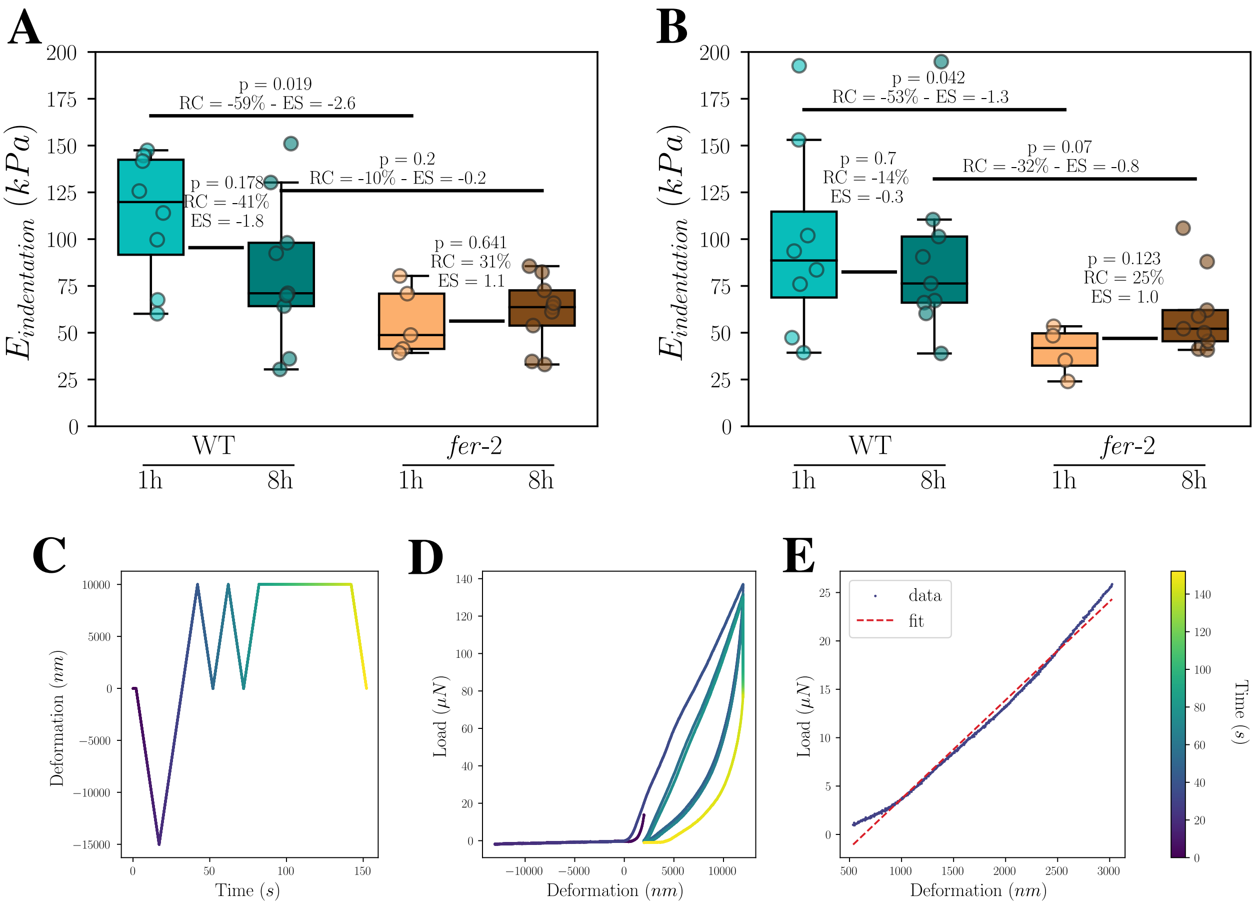

Figure S3: **Microindentation shows a lower elastic modulus in *fer*-2 than in WT.** (**A-B**) Indentation modulus $E_{indentation}$ for WT and *fer*-2 at 1 h and 8 h after imbibition. Measurements were made on the central part of the gemma (**A**) or on the growing periphery (**B**). WT(1 h): n = 8, WT(8 h): n = 9, *fer*-2(1 h): n = 5, *fer*-2(8 h): n = 9. (**C-E**) Example of an indentation curve. The set displacement (**C**), an example of a whole load response to the set deformation (**D**) and the portion of the curve fitted to extract the modulus (**E**). The time is colour-coded for the curves.

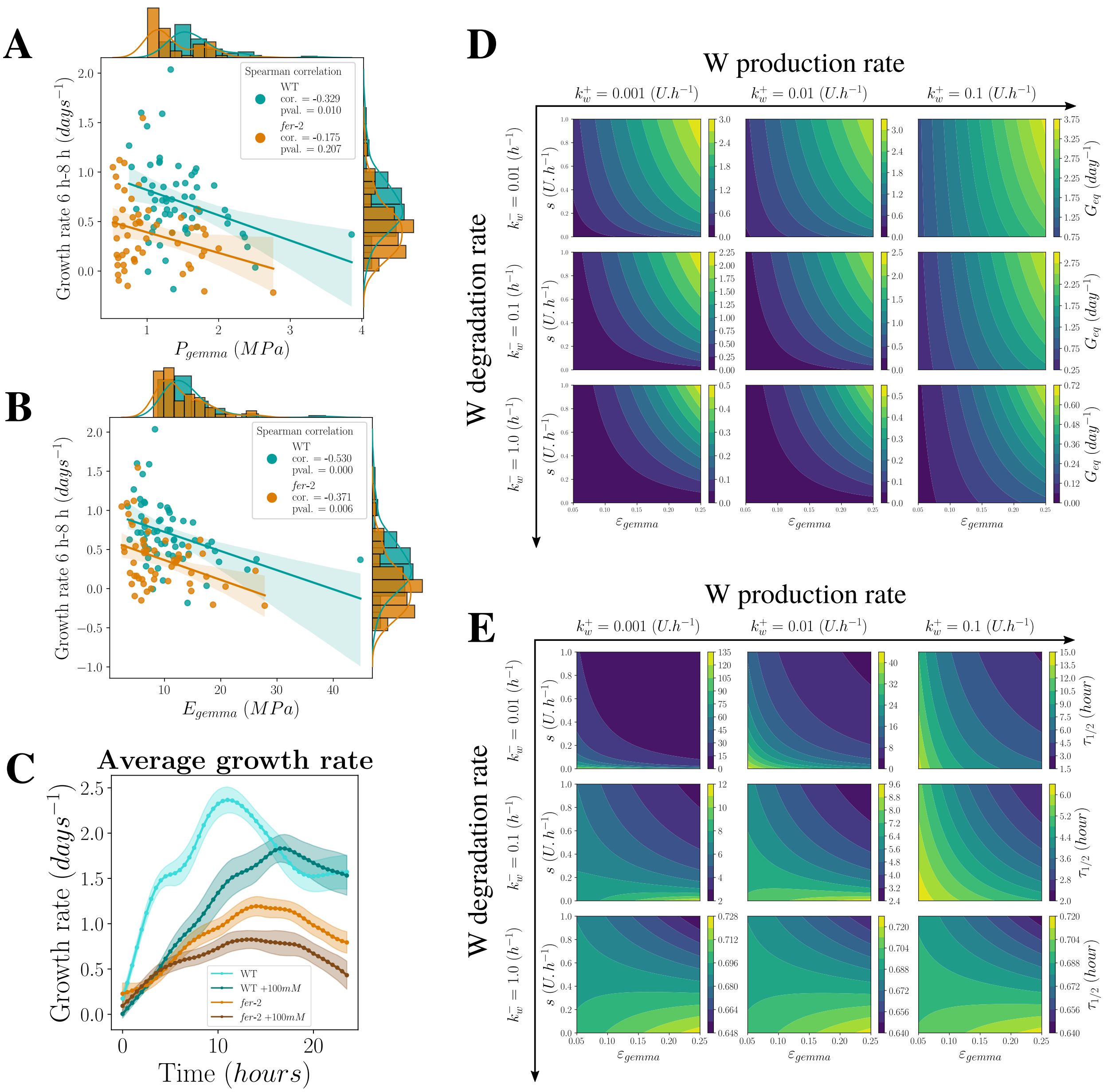
A
Spearman correlation
WT
cor. = -0.329
pval. = 0.010
fer-2
cor. = -0.175
pval. = 0.207
Growth rate 6 h-8 h ($days^{-1}$)
P_{gemma} (MPa)
B
Spearman correlation
WT
cor. = -0.530
pval. = 0.000
fer-2
cor. = -0.371
pval. = 0.006
Growth rate 6 h-8 h ($days^{-1}$)
E_{gemma} (MPa)
C
Average growth rate
Growth rate ($days^{-1}$)
Time ($hours$)
WT
WT +100mM
fer-2
fer-2 +100mM
D
W production rate
$k_w^+ = 0.001$ ($U.h^{-1}$)
$k_w^+ = 0.01$ ($U.h^{-1}$)
$k_w^+ = 0.1$ ($U.h^{-1}$)
W degradation rate
$k_w^- = 0.01$ (h^{-1})
$k_w^- = 0.1$ (h^{-1})
$k_w^- = 1.0$ (h^{-1})
s ($U.h^{-1}$)
ε_{gemma}
G_{eq} (day^{-1})
E
W production rate
$k_w^+ = 0.001$ ($U.h^{-1}$)
$k_w^+ = 0.01$ ($U.h^{-1}$)
$k_w^+ = 0.1$ ($U.h^{-1}$)
W degradation rate
$k_w^- = 0.01$ (h^{-1})
$k_w^- = 0.1$ (h^{-1})
$k_w^- = 1.0$ (h^{-1})
s ($U.h^{-1}$)
ε_{gemma}
$\tau_{1/2}$ ($hour$)

Figure S4: **FERONIA regulates extensibility** (**A-B**) Correlation analysis between instantaneous growth rate averaged between 6 h and 8 h post imbibition and mechanical parameters (**A**) turgor P_{gemma} and (**B**) elastic modulus E_{gemma} for both WT and *fer*-2. Scatter plot and regression line with 95% confident interval are represented as well as histograms per genotype for each variable. *cor.* stands for the Spearman correlation coefficient. WT: n = 62, rep. = 3 and *fer*-2: n = 56, rep. = 3. (**C**) Average growth rates for WT and *fer*-2, with and without osmotic treatment (+100 mM or +0 mM mannitol). Shaded areas correspond to the 95% confident interval. WT(+0 mM): n = 193, rep. = 4. WT(+100 mM): n = 136, rep. = 3. *fer*-2(+0 mM): n = 69, rep. = 6. *fer*-2(+100 mM): n = 70, rep. = 3. (**D-E**) Parameters space exploration for the mathematical model for different values of the W production rate k_w^+ and degradation rate k_w^-. Model output according to the sensing parameter s and the elastic deformation ε_{gemma} for (**D**) the equilibrium growth rate G_{eq} and (**E**) the germination characteristic time $\tau_{1/2}$. Other parameters are set to: $t_s = 1\ h$, $\beta = 0.5\ U$, $E = 10\ MPa$ and $Y = 0.01$.

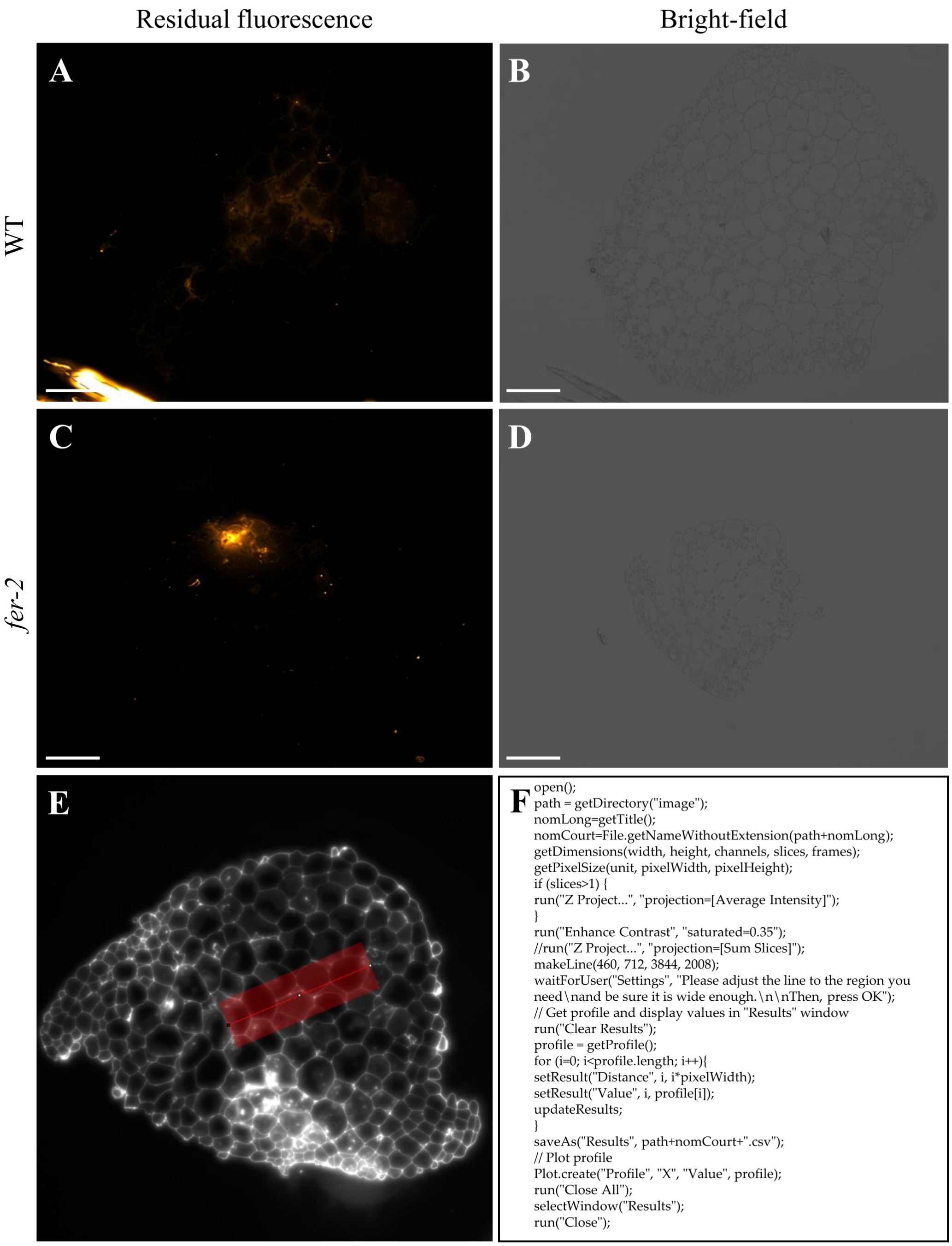


```
open();
path = getDirectory("image");
nomLong=getTitle();
nomCourt=File.getNameWithoutExtension(path+nomLong);
getDimensions(width, height, channels, slices, frames);
getPixelSize(unit, pixelWidth, pixelHeight);
if (slices>1) {
run("Z Project...", "projection=[Average Intensity]");
}
run("Enhance Contrast", "saturated=0.35");
//run("Z Project...", "projection=[Sum Slices]");
makeLine(460, 712, 3844, 2008);
waitForUser("Settings", "Please adjust the line to the region you
need\nand be sure it is wide enough.\n\nThen, press OK");
// Get profile and display values in "Results" window
run("Clear Results");
profile = getProfile();
for (i=0; i<profile.length; i++){
setResult("Distance", i, i*pixelWidth);
setResult("Value", i, profile[i]);
updateResults;
}
saveAs("Results", path+nomCourt+".csv");
// Plot profile
Plot.create("Profile", "X", "Value", profile);
run("Close All");
selectWindow("Results");
run("Close");
```

Figure S5: **Immunolabelling negative controls of Marchantia gemmae after embedding and region of interest (ROI) defined for each pictures of Marchantia and ImageJ macro.** Negative controls samples in which primary antibodies were omitted treated only with secondary antibody coupled Alexa647 show no fluorescence in (**A,B**) WT and (**C,D**) *fer*-2 gemmae. (**E**) The red area corresponds to the fluorescent measurement area. (**F**) To automate the fluorescent measurements, an ImageJ macro developed for fluorescence measurements. Scale bars are 100 µm.

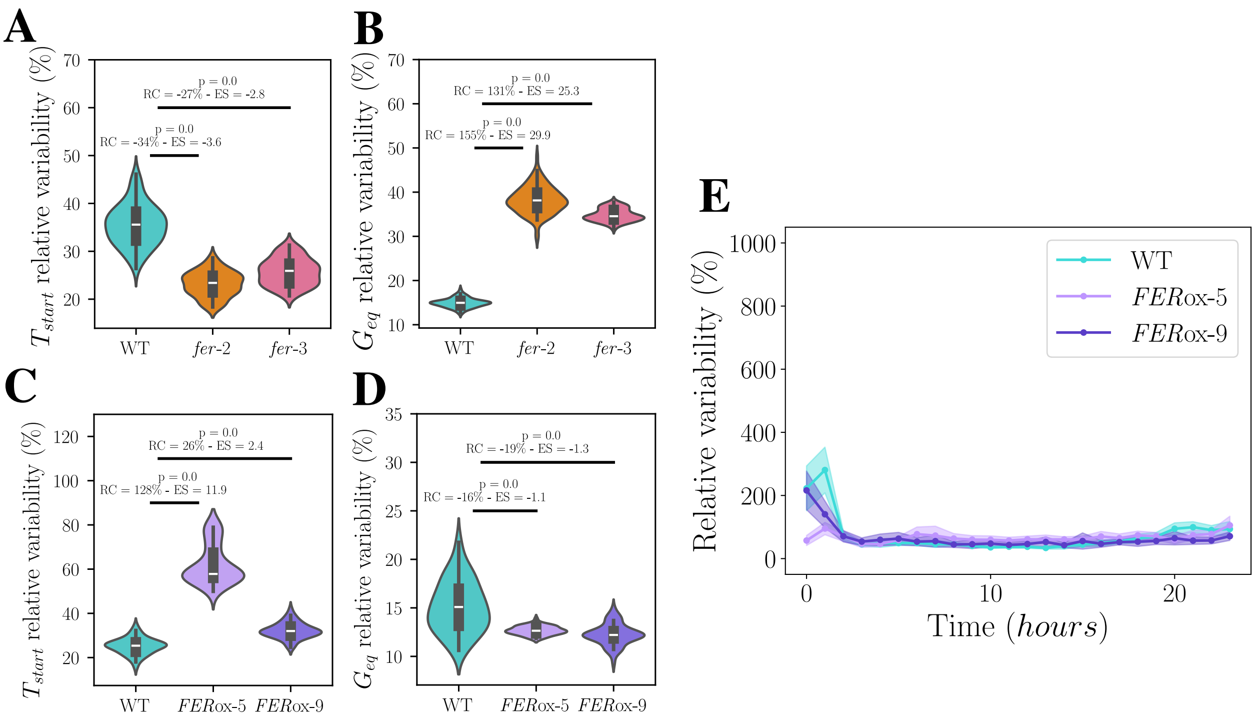

Figure S6: **FERONIA regulates growth variability.** (**A-B**) Relative variability estimated by relative average absolute deviation of the experimental growth parameters for *fer*-2, *fer*-3 and WT for (**A**) the germination time T_{start} and (**B**) the equilibrium growth rate G_{eq}. WT: n = 178 and rep. = 5. *fer*-2: n = 141 and rep. = 3. *fer*-3: n = 104 and rep. = 3. (**A-B**) Violin plot of the relative variability in experiments (estimated by the average absolute deviation normalised by the mean) of WT, *FER*ox-5 and *FER*ox-9 for (**A**) the germination time T_{start} and (**B**) the equilibrium growth rate G_{eq}. WT: n = 190 individuals, rep. = 6. *FER*ox-5: n = 52 individuals, rep. = 3. *FER*ox-9: n = 57 individuals and rep. = 3. (**E**) Quantification of the variability (AAD over the median) of the local growth rate during 24h of growth, for WT, *FER*ox-5 and *FER*ox-9. Shaded areas represent the 95% confident interval. WT: n = 84 and rep. = 4. *FER*ox-5: n = 32 individuals, rep. = 3. *FER*ox-9: n = 39 individuals and rep. = 3.

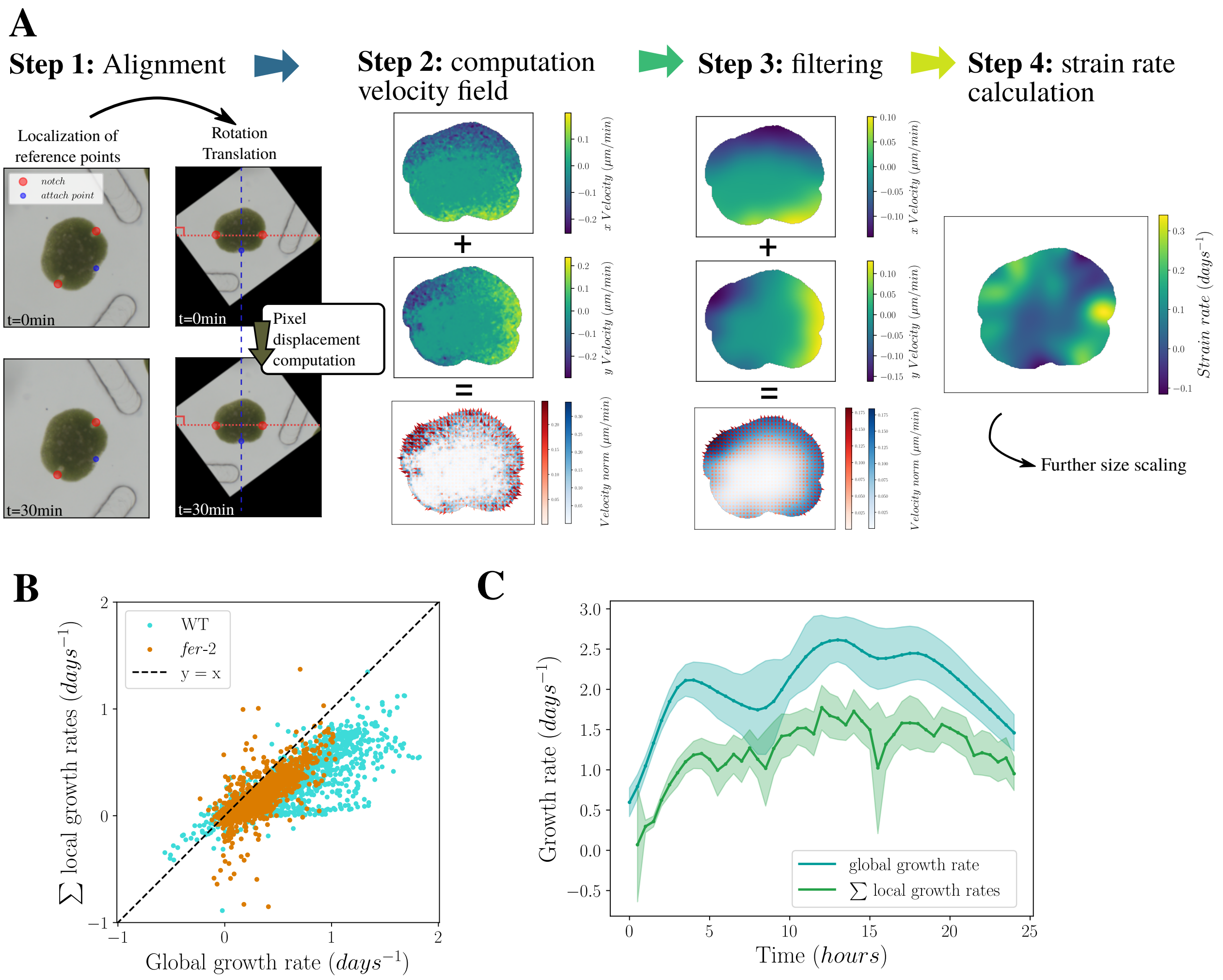

Figure S7: **Method to compute to local displacements (to obtain local deformation or local growth maps).** (**A**) Steps of the strain rate computation method, details in the method (the gemma taken as an example is the WT gemma of Fig. 1-B). (**B**) Comparison of the global growth rate measured by $\frac{1}{A}\frac{\partial A}{\partial t}$ and the sum of the local growth rates during a time step ∂t for growth experiments in chip with different genotypes (WT and *fer*-2). 48 time steps between 0 h and 24 h taken into account. WT: n = 22, rep. = 1. *fer*-2: n = 24, rep. = 1. (**C**) Comparison of the global growth rate dynamics and the sum of the local growth rates for a given WT growth experiment in a chip. n = 21, rep. = 1. Shaded areas represent the 95% confident interval.

Table S1: Parameters estimates for the model.

**Parameter**	**Estimated value (unit)**	**References**
P - turgor pressure	0.5-2.5 (MPa)	this work (osmotic steps measurements)
Y - yielding threshold	few	this work (deduction from growth arrest)
E - elastic modulus	2-20 (MPa)	this work (osmotic steps measurements)
Φ_w - molecular extensibility	$0,36$ ($h^{-1}.U^{-1}$)	(*79*) adapted to the formulation with ε_{gemma}
k_w^+ - W production rate	($U.h^{-1}$)	arbitrary units
k_w^- - W degradation rate	($hours^{-1}$ to $days^{-1}$)	(*80*)

Table S2: Details of the epitopes recognised by the monoclonal antibodies used in the study.

**Cell Wall Glycomolecules**		**mAb/probes**	**Epitope**	**References**
Cellulose		Direct Red 23	Crystalline cellulose	Anderson et al., 2010 (*42*), Liesche et al., 2023 (*81*)
Hemicellulose	Xyloglucan	LM15	Xylosylated xyloglucan (XXXG)	Marcus et al., 2008 (*82*), Rydahl et al., 2018 (*83*)
		LM24	Galactosylated xyloglucan (XXLG)	Pedersen et al., 2012 (*84*), Rydahl et al., 2018 (*83*)
		LM25	Galactosylated xyloglucan (XXLG, XLLG)	Pedersen et al., 2012 (*84*), Rydahl et al., 2018 (*83*)
	Heteromannan	LM21	$\beta-(1 \rightarrow 4)$-mannan backbone epitope from DP2 to DP5	Ordaz-ortiz et al., 2009 (*85*), Rydahl et al., 2018 (*83*)
Pectin	Homogalacturonan	LM19	Homogalacturonan with low degree of esterification	Verhertbruggen et al., 2009 (*86*), Rydahl et al., 2018 (*83*)
		LM20	Homogalacturonan with high degree of esterification	Verhertbruggen et al., 2009 (*86*), Rydahl et al., 2018 (*83*)
	Rhamnogalacturonan I	LM13	Specific subset of unbranched pectic $(1 \rightarrow 5)-\alpha-L$-arabinan	Moller et al., 2007 (*87*), Rydahl et al., 2018 (*83*)
Proteoglycan	Arabinongalactan proteins	LM2	AGP,$(1 \rightarrow 6)-\beta-D$ galactan chain with terminally attached Glucuronic acid	Yates et al., 1996 (*88*), Ruprecht et al., 2017 (*89*)

Table S3: **Marchantia lines**

**Genotype**	**Description**	**WT back-ground**	**Origin**
Tak-1	WT male Marchantia	-	-
fer-2	*FERONIA* knock-out Crispr/Cas mediated insertion	Tak-1	Mecchia et al., 2022 (*19*)
fer-3	*FERONIA* knock-out Crispr/Cas mediated insertion	Tak-1	Mecchia et al., 2022 (*19*)
*FER*ox-5	*FERONIA* overexpressor - $_{pro}$Mp*EF1*:*FER*-Cit	Tak-1	Mecchia et al., 2022 (*19*)
*FER*ox-9	*FERONIA* overexpressor - $_{pro}$Mp*EF1*:*FER*-Cit	Tak-1	Mecchia et al., 2022 (*19*)

REFERENCES

1. D. J. Cosgrove, Catalysts of plant cell wall loosening. *F1000Res.* **5**, F1000 Faculty Rev–119 (2016).

2. O. Hamant, J. Traas, The mechanics behind plant development. *New Phytol.* **185**, 369–385 (2010).

3. D. J. Cosgrove, Structure and growth of plant cell walls. *Nat. Rev. Mol. Cell Biol.* **25**, 340–358 (2024).

4. O. Hamant, E. S. Haswell, Life behind the wall: Sensing mechanical cues in plants. *BMC Biol.* **15**, 59 (2017).

5. A. Malivert, O. Hamant, Why is FERONIA pleiotropic? *Nat. Plants* **9**, 1018–1025 (2023).

6. A. Y. Cheung, FERONIA: A receptor kinase at the core of a global signaling network. *Annu. Rev. Plant Biol.* **75**, 345–375 (2024).

7. X. Zhang, Z. Yang, D. Wu, F. Yu, RALF–FERONIA signaling: Linking plant immune response with cell growth. *Plant Commun.* **1**, 100084 (2020).

8. S. Li, Y. Zhang, To grow or not to grow: FERONIA has her say. *Mol. Plant* **7**, 1261–1263 (2014).

9. J.-M. Escobar-Restrepo, N. Huck, S. Kessler, V. Gagliardini, J. Gheyselinck, W. C. Yang, U. Grossniklaus, The FERONIA receptor-like kinase mediates male-female interactions during pollen tube reception. *Science* **317**, 656–660 (2007).

10. F. Yu, J. Li, Y. Huang, L. Liu, D. Li, L. Chen, S. Luan, FERONIA receptor kinase controls seed size in *Arabidopsis thaliana*. *Mol. Plant* **7**, 920–922 (2014).

11. M. Haruta, G. Sabat, K. Stecker, B. B. Minkoff, M. R. Sussman, A peptide hormone and its receptor protein kinase regulate plant cell expansion. *Science* **343**, 408–411 (2014).

12. K. Dünser, S. Gupta, A. Herger, M. I. Feraru, C. Ringli, J. Kleine-Vehn, Extracellular matrix sensing by FERONIA and leucine-rich repeat extensins controls vacuolar expansion during cellular elongation in *Arabidopsis thaliana*. *EMBO J.* **38**, e100353 (2019).

13. H. Guo, L. Li, H. Ye, X. Yu, A. Algreen, Y. Yin, Three related receptor-like kinases are required for optimal cell elongation in *Arabidopsis thaliana*. *Proc. Natl. Acad. Sci. U.S.A.* **106**, 7648–7653 (2009).

14. S. D. Deslauriers, P. B. Larsen, FERONIA is a key modulator of brassinosteroid and ethylene responsiveness in *Arabidopsis* hypocotyls. *Mol. Plant* **3**, 626–640 (2010).

15. S. A. Kessler, H. Shimosato-Asano, N. F. Keinath, S. E. Wuest, G. Ingram, R. Panstruga, U. Grossniklaus, Conserved molecular components for pollen tube reception and fungal invasion. *Science* **330**, 968–971 (2010).

16. Q. Duan, D. Kita, C. Li, A. Y. Cheung, H.-M. Wu, FERONIA receptor-like kinase regulates RHO GTPase signaling of root hair development. *Proc. Natl. Acad. Sci. U.S.A.* **107**, 17821–17826 (2010).

17. S. Schoenaers, H. K. Lee, M. Gonneau, E. Faucher, T. Levasseur, E. Akary, N. Claeijs, S. Moussu, C. Broyart, D. Balcerowicz, H. AbdElgawad, A. Bassi, D. S. C. Damineli, A. Costa, J. A. Feijó, C. Moreau, E. Bonnin, B. Cathala, J. Santiago, H. Höfte, K. Vissenberg, Rapid alkalinization factor 22 has a structural and signalling role in root hair cell wall assembly. *Nat. Plants* **10**, 494–511 (2024).

18. H.-W. Shih, N. D. Miller, C. Dai, E. P. Spalding, G. B. Monshausen, The receptor-like kinase FERONIA is required for mechanical signal transduction in *Arabidopsis* seedlings. *Curr. Biol.* **24**, 1887–1892 (2014).

19. M. A. Mecchia, M. Rövekamp, A. Giraldo-Fonseca, D. Meier, P. Gadient, H. Vogler, D. Limacher, J. L. Bowman, U. Grossniklaus, *The single Marchantia polymorpha FERONIA homolog reveals an ancestral role in regulating cellular expansion and integrity* **149**, dev200580 (2022).

20. K. Hématy, P. E. Sado, A. van Tuinen, S. Rochange, T. Desnos, S. Balzergue, S. Pelletier, J. P. Renou, H. Höfte, A receptor-like kinase mediates the response of *Arabidopsis* cells to the inhibition of cellulose synthesis. *Curr. Biol.* **17**, 922–931 (2007).

21. W. Feng, D. Kita, A. Peaucelle, H. N. Cartwright, V. Doan, Q. Duan, M. C. Liu, J. Maman, L. Steinhorst, I. Schmitz-Thom, R. Yvon, J. Kudla, H. M. Wu, A. Y. Cheung, J. R. Dinneny, The FERONIA receptor kinase maintains cell-wall integrity during salt stress through Ca^{2+} signaling. *Curr. Biol.* **28**, 666–675.e5 (2018).

22. W. Lin, W. Tang, X. Pan, A. Huang, X. Gao, C. T. Anderson, Z. Yang, *Arabidopsis* pavement cell morphogenesis requires FERONIA binding to pectin for activation of ROP GTPase signaling. *Curr. Biol.* **32**, 497–507.e4 (2022).

23. D. Ji, T. Chen, Z. Zhang, B. Li, S. Tian, Versatile roles of the receptor-like kinase Feronia in plant growth, development and host-pathogen interaction. *Int. J. Mol. Sci.* **21**, 7881 (2020).

24. N. F. Keinath, S. Kierszniowska, J. Lorek, G. Bourdais, S. A. Kessler, H. Shimosato-Asano, U. Grossniklaus, W. X. Schulze, S. Robatzek, R. Panstruga, PAMP (pathogen-associated molecular pattern)-induced changes in plasma membrane compartmentalization reveal novel components of plant immunity*. *J. Biol. Chem.* **285**, 39140–39149 (2010).

25. L. Vaahtera, J. Schulz, T. Hamann, Cell wall integrity maintenance during plant development and interaction with the environment. *Nat. Plants* **5**, 924–932 (2019).

26. A. Malivert, Ö. Erguvan, A. Chevallier, A. Dehem, R. Friaud, M. Liu, M. Martin, T. Peyraud, O. Hamant, S. Verger, FERONIA and microtubules independently contribute to mechanical integrity in the *Arabidopsis* shoot. *PLOS Biol.* **19**, e3001454 (2021).

27. N. Hervieux, M. Dumond, A. Sapala, A. L. Routier-Kierzkowska, D. Kierzkowski, A. H. K. Roeder, R. S. Smith, A. Boudaoud, O. Hamant, A mechanical feedback restricts sepal growth and shape in *Arabidopsis*. *Curr. Biol.* **26**, 1019–1028 (2016).

28. O. Hamant, M. G. Heisler, H. Jönsson, P. Krupinski, M. Uyttewaal, P. Bokov, F. Corson, P. Sahlin, A. Boudaoud, E. M. Meyerowitz, Y. Couder, J. Traas, Developmental patterning by mechanical signals in *Arabidopsis*. *Science* **322**, 1650–1655 (2008).

29. B. Bozorg, P. Krupinski, H. Jönsson, Stress and strain provide positional and directional cues in development. *PLOS Comput. Biol.* **10**, e1003410 (2014).

30. A. Creff, O. Ali, C. Bied, V. Bayle, G. Ingram, B. Landrein, Evidence that endosperm turgor pressure both promotes and restricts seed growth and size. *Nat. Commun.* **14**, 67 (2023).

31. H. Höfte, The Yin and Yang of cell wall integrity control: Brassinosteroid and FERONIA signaling. *Plant Cell Physiol.* **56**, 224–231 (2015).

32. T. H. Yeats, H. Sorek, D. E. Wemmer, C. R. Somerville, Cellulose deficiency is enhanced on hyper accumulation of sucrose by a H^+-coupled sucrose symporter. *Plant Physiol.* **171**, 110–124 (2016).

33. N. Gigli-Bisceglia, E. van Zelm, W. Huo, J. Lamers, C. Testerink, *Arabidopsis* root responses to salinity depend on pectin modification and cell wall sensing. *Development* **149**, dev200363 (2022).

34. C. Liu, H. Yu, A. Voxeur, X. Rao, R. A. Dixon, FERONIA and wall-associated kinases coordinate defense induced by lignin modification in plant cell walls. *Sci. Adv.* **9**, eadf7714 (2023).

35. L. Bacete, J. Schulz, T. Engelsdorf, Z. Bartosova, L. Vaahtera, G. Yan, J. M. Gerhold, T. Tichá, C. Øvstebø, N. Gigli-Bisceglia, S. Johannessen-Starheim, J. Margueritat, H. Kollist, T. Dehoux, S. A. M. McAdam, T. Hamann, THESEUS1 modulates cell wall stiffness and abscisic acid production in *Arabidopsis thaliana*. *Proc. Natl. Acad. Sci. U.S.A.* **119**, e2119258119 (2022).

36. H. Kato, Y. Yasui, K. Ishizaki, Gemma cup and gemma development in *Marchantia polymorpha*. *New Phytol.* **228**, 459–465 (2020).

37. V. Laplaud, E. Muller, N. Demidova, S. Drevensek, A. Boudaoud, Assessing the hydromechanical control of plant growth. *J. R. Soc. Interface* **21**, 20240008 (2024).

38. M. Shimamura, *Marchantia polymorpha*: Taxonomy, phylogeny and morphology of a model system. *Plant Cell Physiol.* **57**, 230–256 (2016).

39. H. Kolkas, V. Burlat, E. Jamet, Immunochemical identification of the main cell wall polysaccharides of the early land plant *Marchantia polymorpha*. *Cells* **12**, 1833 (2023).

40. H. S. Kang, X. Tong, A. Mariette, M. Leong, C. Beahan, E. Flores-Sandoval, G. B. Pedersen, C. Rautengarten, J. L. Bowman, B. Ebert, A. Bacic, M. S. Doblin, S. Persson, E. R. Lampugnani, Cell wall-related glycosyltransferases and wall architecture in the model liverwort *Marchantia polymorpha*. *Plant J.* **123**, e70439 (2025).

41. M. J. Peña, A. G. Darvill, S. Eberhard, W. S. York, M. A. O'Neill, Moss and liverwort xyloglucans contain galacturonic acid and are structurally distinct from the xyloglucans synthesized by hornworts and vascular plants*. *Glycobiology* **18**, 891–904 (2008).

42. C. T. Anderson, A. Carroll, L. Akhmetova, C. Somerville, Real-time imaging of cellulose reorientation during cell wall expansion in *Arabidopsis* roots. *Plant Physiol.* **152**, 787–796 (2010).

43. Y. Zhang, J. Yu, X. Wang, D. M. Durachko, S. Zhang, D. J. Cosgrove, Molecular insights into the complex mechanics of plant epidermal cell walls. *Science* **372**, 706–711 (2021).

44. J. A. Lockhart, An analysis of irreversible plant cell elongation. *J. Theor. Biol.* **8**, 264–275 (1965).

45. E. R. Rojas, S. Hotton, J. Dumais, Chemically mediated mechanical expansion of the pollen tube cell wall. *Biophys. J.* **101**, 1844–1853 (2011).

46. R. Rollin, J.-F. Joanny, P. Sens, Physical basis of the cell size scaling laws. *eLife* **12**, e82490 (2023).

47. A. Fruleux, S. Verger, A. Boudaoud, Feeling stressed or strained? A biophysical model for cell wall mechanosensing in plants. *Front. Plant Sci.* **10**, 757 (2019).

48. N. Hervieux, S. Tsugawa, A. Fruleux, M. Dumond, A. L. Routier-Kierzkowska, T. Komatsuzaki, A. Boudaoud, J. C. Larkin, R. S. Smith, C. B. Li, O. Hamant, Mechanical shielding of rapidly growing cells buffers growth heterogeneity and contributes to organ shape reproducibility. *Curr. Biol.* **27**, 3468–3479.e4 (2017).

49. A. Sampathkumar, P. Krupinski, R. Wightman, P. Milani, A. Berquand, A. Boudaoud, O. Hamant, H. Jönsson, E. M. Meyerowitz, Subcellular and supracellular mechanical stress prescribes cytoskeleton behavior in *Arabidopsis* cotyledon pavement cells. *eLife* **3**, e01967 (2014).

50. M. Uyttewaal, A. Burian, K. Alim, B. Landrein, D. Borowska-Wykręt, A. Dedieu, A. Peaucelle, M. Ludynia, J. Traas, A. Boudaoud, D. Kwiatkowska, O. Hamant, Mechanical stress acts via katanin to amplify differences in growth rate between adjacent cells in *Arabidopsis*. *Cell* **149**, 439–451 (2012).

51. L. Hong, M. Dumond, S. Tsugawa, A. Sapala, A. L. Routier-Kierzkowska, Y. Zhou, C. Chen, A. Kiss, M. Zhu, O. Hamant, R. S. Smith, T. Komatsuzaki, C. B. Li, A. Boudaoud, A. H. K. Roeder, Variable cell growth yields reproducible organ development through spatiotemporal averaging. *Dev. Cell* **38**, 15–32 (2016).

52. A. Boisson-Dernier, C. M. Franck, D. S. Lituiev, U. Grossniklaus, Receptor-like cytoplasmic kinase maris functions downstream of CrRLK1L-dependent signaling during tip growth. *Proc. Natl. Acad. Sci. U.S.A.* **112**, 12211–12216 (2015).

53. J. Westermann, S. Streubel, C. M. Franck, R. Lentz, L. Dolan, A. Boisson-Dernier, An evolutionarily conserved receptor-like kinases signaling module controls cell wall integrity during tip growth. *Curr. Biol.* **29**, 3899–3908.e3 (2019).

54. X. Wang, J. Liu, M. Wang, L. Liu, X. Liu, C. Zhao, FERONIA controls ABA-mediated seed germination via the regulation of CARK1 kinase activity. *Cell Rep.* **43**, 114843 (2024).

55. D. M. Eklund, M. Kanei, E. Flores-Sandoval, K. Ishizaki, R. Nishihama, T. Kohchi, U. Lagercrantz, R. P. Bhalerao, Y. Sakata, J. L. Bowman, An evolutionarily conserved abscisic acid signaling pathway regulates dormancy in the liverwort *Marchantia polymorpha*. *Curr. Biol.* **28**, 3691–3699.e3 (2018).

56. A. Boisson-Dernier, S. Roy, K. Kritsas, M. A. Grobei, M. Jaciubek, J. I. Schroeder, U. Grossniklaus, Disruption of the pollen-expressed FERONIA homologs ANXUR1 and ANXUR2 triggers pollen tube discharge. *Development* **136**, 3279–3288 (2009).

57. E. Li, G. Wang, Y.-L. Zhang, Z. Kong, S. Li, FERONIA mediates root nutating growth. *Plant J.* **104**, 1105–1116 (2020).

58. D. J. Cosgrove, Building an extensible cell wall. *Plant Physiol.* **189**, 1246–1277 (2022).

59. A. J. Bidhendi, A. Geitmann, Relating the mechanics of the primary plant cell wall to morphogenesis. *J. Exp. Bot.* **67**, 449–461 (2016).

60. E. Muller, S. Drevensek, A. Boudaoud, Regulation of plant growth by polysaccharide synthesis and incorporation into the cell wall. *J. Exp. Bot.* **77**, 101–119 (2026).

61. K. Vissenberg, H. Höfte, *Cell Wall-Related Mechanisms Underlying Plant Cell Expansion* (CRC Press, Boca Raton, ed. 1, 2023), pp. 127–146.

62. T. O. Jobe, C. G. L. Junior, S. C. Stolze, L. Stephan, J. Westermann, A. Harzen, M. Hülskamp, H. Nakagami, A. Boisson-Dernier, Multiomics analyses of mutants for *Marchantia polymorpha* FERONIA and MARIS reveal a link between cell wall integrity and abscisic acid responses. *Plant Cell Physiol.* , pcag015 (2026).

63. P. Wang, N. M. Clark, T. M. Nolan, G. Song, P. M. Bartz, C. Y. Liao, C. Montes-Serey, E. Katz, J. K. Polko, J. J. Kieber, D. J. Kliebenstein, D. C. Bassham, J. W. Walley, Y. Yin, H. Guo, Integrated omics reveal novel functions and underlying mechanisms of the receptor kinase FERONIA in *Arabidopsis thaliana*. *Plant Cell* **34**, 2594–2614 (2022).

64. F. B. Daher, L. Serra, R. Carter, H. Jönsson, S. Robinson, E. M. Meyerowitz, W. M. Gray, Xyloglucan deficiency leads to a reduction in turgor pressure and changes in cell wall properties, affecting early seedling establishment. *Curr. Biol.* **34**, 2094–2106.e6 (2024).

65. Y. B. Park, D. J. Cosgrove, Changes in cell wall biomechanical properties in the xyloglucan-deficient *Xxt1/Xxt2* mutant of *Arabidopsis*. *Plant Physiol.* **158**, 465–475 (2012).

66. E. E. Sowinski, B. M. Westman, C. R. Redmond, Y. Kong, A. T. Olek, J. Olek, M. C. McCann, N. C. Carpita, Lack of xyloglucan in the cell walls of the *Arabidopsis* xxt1/xxt2 mutant results in specific increases in homogalacturonan and glucomannan. *Plant J.* **110**, 212–227 (2022).

67. F. Zhao, W. Chen, J. Sechet, M. Martin, S. Bovio, C. Lionnet, Y. Long, V. Battu, G. Mouille, F. Monéger, J. Traas, Xyloglucans and microtubules synergistically maintain meristem geometry and phyllotaxis. *Plant Physiol.* **181**, 1191–1206 (2019).

68. E. Miedes, D. Suslov, F. Vandenbussche, K. Kenobi, A. Ivakov, D. van der Straeten, E. P. Lorences, E. J. Mellerowicz, J. P. Verbelen, K. Vissenberg, Xyloglucan endotransglucosylase/hydrolase (XTH) overexpression affects growth and cell wall mechanics in etiolated *Arabidopsis* hypocotyls. *J. Exp. Bot.* **64**, 2481–2497 (2013).

69. C. T. Anderson, J. Pelloux, The dynamics, degradation, and afterlives of pectins: Influences on cell wall assembly and structure, plant development and physiology, agronomy, and biotechnology. *Annu. Rev. Plant Biol.* **76**, 85–113 (2025).

70. D. Kierzkowski, N. Nakayama, A. L. Routier-Kierzkowska, A. Weber, E. Bayer, M. Schorderet, D. Reinhardt, C. Kuhlemeier, R. S. Smith, Elastic domains regulate growth and organogenesis in the plant shoot apical meristem. *Science* **335**, 1096–1099 (2012).

71. X. Liu, W. Jiang, Y. Li, H. Nie, L. Cui, R. Li, L. Tan, L. Peng, C. Li, J. Luo, M. Li, H. Wang, J. Yang, B. Zhou, P. Wang, H. Liu, J. K. Zhu, C. Zhao, FERONIA coordinates plant growth and salt tolerance via the phosphorylation of phyB. *Nat. Plants* **9**, 645–660 (2023).

72. M.-C. J. Liu, F. L. J. Yeh, R. Yvon, K. Simpson, S. Jordan, J. Chambers, H. M. Wu, A. Y. Cheung, Extracellular pectin-RALF phase separation mediates FERONIA global signaling function. *Cell* **187**, 312–330.e22 (2024).

73. S. Schoenaers, K. Vissenberg, Overlooked aspects of CrRLK1L–RALF signaling. *New Phytol.* **248**, 549–554 (2025).

74. L. Li, H. Chen, S. S. Alotaibi, A. Pěnčík, M. Adamowski, O. Novák, J. Friml, RALF1 peptide triggers biphasic root growth inhibition upstream of auxin biosynthesis. *Proc. Natl. Acad. Sci.* **119**, e2121058119 (2022).

75. K. Abley, R. Goswami, J. C. W. Locke, Bet-hedging and variability in plant development: Seed germination and beyond. *Philos. Trans. R. Soc. B Lond. B Biol. Sci.* **379**, 20230048 (2024).

76. U. Alon, *An Introduction to Systems Biology: Design Principles of Biological Circuits* (Chapman and Hall/CRC, 2019).

77. I. Boulogne, P. Petit, L. Desfontaines, G. Durambur, C. Deborde, C. Mirande-Ney, Q. Arnaudin, C. Plasson, J. Grivotte, C. Chamot, S. Bernard, G. Loranger-Merciris, Biological and chemical characterization of *Musa Paradisiaca* leachate. *Biology* **12**, 1326 (2023).

78. C. Mirande-Ney, Q. Arnaudin, G. Durambur, C. Plasson, S. Bernard, C. Chamot, J. Grivotte, N. Mati-Baouche, A. Driouich, J. Brebion, F. Hennequart, P. Lerouge, I. Boulogne, LAM2: An unusual laminaran structure for a novel plant elicitor candidate. *Biomolecules* **13**, 1483 (2023).

79. H. Nonami, J. S. Boyer, Wall extensibility and cell hydraulic conductivity decrease in enlarging stem tissues at low water potentials. *Plant Physiol.* **93**, 1610–1619 (1990).

80. L. Li, C. J. Nelson, J. Trösch, I. Castleden, S. Huang, A. H. Millar, Protein degradation rate in *Arabidopsis thaliana* leaf growth and development. *Plant Cell* **29**, 207–228 (2017).

81. J. Liesche, I. Ziomkiewicz, A. Schulz, Super-resolution imaging with pontamine fast scarlet 4BS enables direct visualization of cellulose orientation and cell connection architecture in onion epidermis cells. *BMC Plant Biol.* **13**, 226 (2013).

82. S. E. Marcus, Y. Verhertbruggen, C. Hervé, J. J. Ordaz-Ortiz, V. Farkas, H. L. Pedersen, W. G. T. Willats, J. P. Knox, Pectic homogalacturonan masks abundant sets of xyloglucan epitopes in plant cell walls. *BMC Plant Biol.* **8**, 60 (2008).

83. M. G. Rydahl, A. R. Hansen, S. K. Kračun, J. Mravec, Report on the current inventory of the toolbox for plant cell wall analysis: Proteinaceous and small molecular probes. *Front. Plant Sci.* **9**, 581 (2018).

84. H. L. Pedersen, J. U. Fangel, B. McCleary, C. Ruzanski, M. G. Rydahl, M. C. Ralet, V. Farkas, L. von Schantz, S. E. Marcus, M. C. F. Andersen, R. Field, M. Ohlin, J. P. Knox, M. H. Clausen, W. G. T. Willats, Versatile high resolution oligosaccharide microarrays for plant glycobiology and cell wall research*. *J. Biol. Chem.* **287**, 39429–39438 (2012).

85. J. J. Ordaz-Ortiz, S. E. Marcus, J. P. Knox, Cell wall microstructure analysis implicates hemicellulose polysaccharides in cell adhesion in tomato fruit pericarp parenchyma. *Mol. Plant* **2**, 910–921 (2009).

86. Y. Verhertbruggen, S. E. Marcus, A. Haeger, J. J. Ordaz-Ortiz, J. P. Knox, An extended set of monoclonal antibodies to pectic homogalacturonan. *Carbohydr. Res.* **344**, 1858–1862 (2009).

87. I. Moller, I. Sørensen, A. J. Bernal, C. Blaukopf, K. Lee, J. Øbro, F. Pettolino, A. Roberts, J. D. Mikkelsen, J. P. Knox, A. Bacic, W. G. T. Willats, High-throughput mapping of cell-wall polymers within and between plants using novel microarrays. *Plant J.* **50**, 1118–1128 (2007).

88. E. A. Yates, J. F. Valdor, S. M. Haslam, H. R. Morris, A. Dell, W. Mackie, J. P. Knox, Characterization of carbohydrate structural features recognized by anti-arabinogalactan-protein monoclonal antibodies. *Glycobiology* **6**, 131–139 (1996).

89. C. Ruprecht, M. P. Bartetzko, D. Senf, P. Dallabernadina, I. Boos, M. C. F. Andersen, T. Kotake, J. P. Knox, M. G. Hahn, M. H. Clausen, F. Pfrengle, A synthetic glycan microarray

enables epitope mapping of plant cell wall glycan-directed antibodies. *Plant Physiol.* **175**, 1094–1104 (2017).
